# Cyclic assisted cloning of arbitrary unknown single-particle states in amplitude damping channel

**DOI:** 10.1371/journal.pone.0329370

**Published:** 2025-09-02

**Authors:** Nueraminaimu Maihemuti, Yimamujiang Aisan, Jiayin Peng, Zhongwen Wang, Jiangang Tang

**Affiliations:** School of Mathematics and Statistics, Kashi University, Kashi, Xinjiang, China; Universita degli Studi Roma Tre, ITALY

## Abstract

In this paper, two conclusive three-party cyclic assisted cloning protocols in amplitude damping (AD) channel are put forward that, respectively clone three arbitrary unknown single-qubit states and single-qutrit states with the help of a state preparer. Each of our protocols includes three consecutive stages: quantum channel preparation, cyclic quantum teleportation (CQT), and multi-party assisted cloning. The first stage of each protocol proposes the detailed processes of sharing a pure entangled quantum state as a component of a quantum channel in AD channel via entanglement compensation. In second stage, a three-party CQT is presented where three unknown single-qubit states (or single-qutrit states) are reconstructed simultaneously in three different places, respectively, by introducing auxiliary qubits and performing appropriate operations. In the third stage, the state preparer Victor performs one multi-qubit measurement (or one unitary transformation and one multi-qutrit measurement) and informs the three communicators of his outcome, three distinct unknown single-qubit states or their orthogonal complement states (or single-qutrit states) are cloned simultaneously and with probability at three separate locations,respectively. Furthermore, we extend the above protocols from two aspects: (i) the extension to the case of (N+1) participants; (ii) extension to the case of *d*-dimensional unknown single-qudit state cycle-assisted cloning.

## 1 Introduction

The fundamental principles of quantum mechanics, such as quantum parallelism, quantum state superposition, quantum entanglement, and quantum coherence—features with no classical counterparts—demonstrate revolutionary advantages in computing and communication. With shared quantum entanglement and local operation as well as classical communication, in 1993 Bennett et al. [1] first proposed the concept of quantum teleportation (QT), a clever application of quantum mechanics in the field of information, which has attracted great attention because it provides unconditional security in the communication process. Since the seminal work of Bennett et al. [1] was reported, the vast majority of quantum communication branches have developed rapidly, such as quantum key distribution (QKD) [2,3], quantum secure direct communication (QSDC) [4,5], quantum dialogue (QD) [6,7], QT [8–10], quantum secret sharing (QSS) [11,12], remote state preparation (RSP) [13–16], remote implementation of quantum operation (RIQO) [17,18], and so on.

The no-cloning theorem, as another important principle of quantum mechanics, states that it is impossible to perfectly replicate any unknown quantum state in quantum mechanics. This conclusion is proven through the linear superposition property of quantum states using proof by contradiction, and it has profoundly influenced the development of quantum information science [19]. The theoretical breakthroughs and technological innovations addressing this limitation have always been one of the core topics in quantum information science: Bužek and Hillery, by constructing a theoretical model for a universal quantum cloning machine [20–22] first systematically explained the inevitability of errors in the approximate cloning of non-orthogonal states and their quantification, laying the key theoretical foundation for subsequent research. Within this framework, Gisin and Massar [23] and Bruß et al. [24] proposed optimal state-dependent cloning schemes and established theoretical limits on the cloning fidelity of specific quantum state families. Later, Duan and Guo pioneered the probabilistic cloning paradigm [25,26], introducing a measurement-feedback mechanism that successfully enabled the limited-probability exact replication of non-orthogonal quantum states. This breakthrough redefined the impossibility of cloning operations from the deterministic to the probabilistic domain, providing a new methodology for quantum state manipulation. As the theoretical system improved, researchers expanded the application boundaries of cloning protocols from multiple dimensions. In 1999, Murao et al. [27] combined quantum teleportation with the optimal cloning framework to construct a remote cloning protocol with one input and multiple outputs. This work led to the design of specialized quantum cloning machines for different physical constraints [28–31]. In 2000, Pati proposed a state preparer-assisted cloning scheme [32] that uses a two-stage protocol to probabilistically generate both the original state and its orthogonal complement copies(OCC). The core idea was later extended by Zhan [33] to the cloning of two-particle entangled states. Han’s team [34] further improved the protocol’s efficiency by employing generalized quantum measurements, and the robustness of the scheme based on cluster states [35,36] and GHZ-class states [37] provided new ideas for cloning operations in noisy environments. In terms of system dimension expansion, research has progressed from single qubits to multi-particle entangled states [38]and high-dimensional quantum dit systems [39–41]. The works of Chen et al. [40] and the Xue-Jiang team [41] have systematically established a universal framework for cloning multi-qudit states. The current frontier of research focuses on the design of practical cloning protocols under noisy channels. Recent progress includes Maihemuti et al.’s [42] proposal of a controlled cloning scheme for multi-qubit states and Zhai’s team’s [43] single-quantum dit state assisted cloning framework. These achievements present three major development trends: optimizing resource consumption through measurement strategies, expanding system dimensions to higher degrees of freedom, and adaptive designs to address decoherence effects. It is worth noting that although the existing theoretical frameworks have verified the feasibility of cloning protocols under ideal conditions, the dynamic evolution characteristics in practical noisy channels (such as the AD channel) have not been fully revealed. This presents a key innovative entry point for this study’s development of cyclic-assisted cloning mechanisms. The entanglement resource optimization methods [35–37] and measurement feedback techniques [25,26,34] developed in prior work provide the necessary technological foundation for exploring robust cloning protocols in noisy environments.

However, almost all the existing quantum assisted cloning schemes are unidirectional (i.e., the copy or OCC of original unknown state appears only at the sender’s location), and they only focus on the case of an ideal environment . Until now,there has been a lack of a multi-directional cloning scheme in a noisy environment, i.e., a scheme that is valid for open systems and simultaneously clones different unknown states at the positions of multiple participants, separately. Recent innovations in quantum communication protocol architectures have shown a significant trend of multidirectional expansion. Researchers are continuously breaking the performance boundaries of traditional schemes by optimizing entanglement resources and measurement strategies. Yang et al. [44] constructed a bidirectional quantum teleportation protocol for three-particle GHZ states based on controlled-NOT(CNOT) operations and single-qubit projective measurements, and extended it to multi-qubit *GHZ* states. Notably, they effectively suppressed the deterioration of teleportation fidelity caused by quantum noise using weak measurements and their inverse operations, providing an important example for managing entanglement resources in noisy channels. In the field of state preparation, Ref. [45] established a bidirectional RSP framework for two arbitrary single-particle states in an ideal environment, further extending it to multi-particle states and verifying the enhancement mechanism of weak measurement techniques for fidelity in noisy environments. In response to complex quantum operation transmission requirements, Ref. [46] designed a remote implementation (RI) protocol for unknown operations with multiple controllers, successfully achieving distributed collaborative execution of multiple partially unknown operations (PUOs). Notably, a series of breakthroughs have been made in the construction of cyclic quantum communication architectures: the Sun-Zhang team [47] implemented a four-node controlled CQT (CCQT) protocol using multi-particle partially entangled states, and their design concept, which can be extended to N-node quantum networks, provides a key architectural reference for future distributed quantum systems. The Peng and Lei scheme [48] built a cyclic remote state preparation protocol using a six-particle entangled channel and achieved bidirectional cyclic operations for N communication nodes through channel reconstruction. Refs. [49,50] proposed bidirectional quantum communication protocols based on a thirteen-particle entangled channel and achieved collaborative architectures for cyclic quantum state transmission and remote execution of PUOs under controlled conditions. In the area of noise adaptability, Ref. [51] innovatively constructed a multiparty quantum state sharing protocol under AD channels, achieving controllable distribution of single-qubit and single-qutrit states, and the noise compensation strategy it employed provides a new methodology for the design of open quantum system communication protocols. These advances collectively highlight the enhancement of channel robustness through the introduction of noise suppression technologies such as weak measurement, the scalability of protocols utilizing high-dimensional entanglement resources [47–49], and the development of collaborative cyclic operation architectures with multiple participants [47–50]. However, existing cyclic communication protocols largely focus on the basic functions of state transmission and operation execution, while the collaborative mechanism for cyclic-assisted cloning in noisy channels remains to be solved. This provides a key innovative idea for this study to construct a cyclic-assisted cloning protocol for arbitrary single-particle states under AD channels, with the weak measurement compensation techniques, multi-particle entanglement channel optimization methods, and noise-adaptive architectures developed in prior work laying the necessary technical foundation for the protocol design in this paper.

Based on the theoretical foundation and technical insights from the multi-party cyclic communication architecture [47–51], this study investigates the controlled cyclic cloning of arbitrary unknown single-particle states with the assistance of a state preparer in the AD channel.In order to achieve this, we first propose two conclusive three-party cyclic schemes, respectively cloning three different unknown single-qubit and single-qutrit states with the help of a state preparer. The entire procedure mainly consists of three stages, in order: first, sharing a pure entangled state (PES) among the three communicators via entanglement compensation; second, performing three-party CQT;third, requiring an assisted cloning with three outputs. We then generalize these schemes in two aspects: first, extending them to the case of an arbitrary multi-party cyclic scenario; and second, extending them to the case of cyclic cloning of arbitrary high-dimensional quantum states.

The structure of this article is organized as follows. In Sect 2, we provide a brief review of the concepts closely related to this study. Sect 3 presents a three-party cyclic protocol for the assisted cloning of three different single-particle states in an AD channel and extends it to the case of *N*-party (*N*>3) cyclic assisted cloning. In Sects 4 and 5, we focus on the three-party cyclic schemes in the AD channel, where the cloning of arbitrary unknown three-dimensional quantum states and *d*-dimensional quantum states, respectively. Finally, Sect 6 offers a brief discussion, followed by the conclusions.

## 2 Preliminaries

This section systematically outlines the foundational knowledge relied upon in the quantum state cyclic-assisted cloning protocol. In a two-level quantum system, any single-qubit state can be expressed as a linear superposition of the computational basis vectors |0⟩ and |1⟩, which can be written as:

|φ⟩=α|0⟩+β|1⟩, (1)

where the complex numbers *α* and *β* satisfy $|\alpha {|}^{2}\;+\;|\beta {|}^{2}=1$, and its orthogonal complement state is $|\varphi_{⊥}\rangle =\alpha^{*}|1\rangle -\beta^{*}|0\rangle$.

The foundational operations of quantum computing are built upon the strict mathematical representation of quantum basis vectors and unitary transformations. The computational basis for a single-qubit, |0⟩,|1⟩ is called the *Z*-basis, while the *X*-basis is $\left\{|\;+\;\rangle =(|0\rangle \;+\;|1\rangle )/\sqrt{2},|-\rangle =(|0\rangle -|1\rangle )/\sqrt{2}\right\}$. In the context of entangled state operations, the complete measurement basis for a two-qubit system is usually the Bell basis

$$|{ℬ}_{kl}\rangle =\frac{1}{\sqrt{2}}[(-1{)}^{k}|k,l\rangle \;+\;|1⊕k,1⊕l\rangle ],\;k,l\in \{0,1\}$$ (2)

where the modulo 2 addition ⊕ ensures the orthonormality of the basis vectors.

The core operation set for single-qubit unitary transformations includes the parameterized Pauli operators

$$\sigma^{\left(s,t\right)}=|0\rangle \langle s⊕t|\;+\;(-1{)}^{s}|t\rangle \langle 1⊕s⊕t|,\;s,t=0,1.$$ (3)

Special instances of these operators include: the identity operator $I=\sigma^{\left(0,0\right)}$, the bit-flip operator $\sigma_{x}=\sigma^{\left(0,1\right)}$, the joint phase-bit-flip operator $i\sigma_{y}=\sigma^{\left(1,0\right)}$, and the phase-flip operator $\sigma_{z}=\sigma^{\left(1,1\right)}$.

Basis vector transformation operations are implemented through the Hadamard gate

$$H=\frac{1}{\sqrt{2}}(|0\rangle \langle 0|\;+\;|0\rangle \langle 1|\;+\;|1\rangle \langle 0|-|1\rangle \langle 1|),$$ (4)

which maps the *Z*-basis to the *X*-basis, thereby constructing the quantum state superposition property.

At the level of two-qubit operations, the CNOT gate ${𝒩}_{uv}$, which is defined as follows

$${𝒩}_{uv}|st\rangle_{uv}=|s\rangle |s⊕t\rangle ,$$ (5)

serves as the standard implementation of a conditional logic gate, where particle *u* is the control particle and particle *v* is the target particle. This operation provides the foundational support for entanglement state preparation by establishing correlations between quantum bits.

Similarly, quantum trit (qutrit) in a a three-level quantum system is a superposition state of states |0⟩, |1⟩ and |2⟩:

$$|\psi \rangle =y_{0}|0\rangle \;+\;y_{1}e^{i\theta_{1}}|1\rangle \;+\;y_{2}e^{i\theta_{2}}|2\rangle ,$$ (6)

where all real numbers *y*_0_, *y*_1_ and *y*_2_ satisfy $|y_{0}{|}^{2}\;+\;|y_{1}{|}^{2}\;+\;|y_{2}{|}^{2}=1$, and *θ*_1_, *θ*_2_ are arbitrary real numbers.

The orthogonal bases {|0⟩,|1⟩,|2⟩} and $\left\{|\xi_{j}\rangle =(|0\rangle \;+\;e^{2\pi ij/3}|1\rangle \;+\;e^{4\pi ij/3}|2\rangle )/\sqrt{3}|j=0,1,2\right\}$ are two mutually unbiased measurement bases in a three-level quantum system, where are also called the *Z*-basis and *X*-basis of this quantum system, respectively. A special class of maximally entangled states composed of two qutrits is collectively referred to as generalized Bell states, which are defined as [51]:

$$|{GB}_{st}\rangle =\frac{1}{\sqrt{3}}\sum_{l=0}^{2}e^{2\pi isl/3}|l,(l\;+\;t)mod3\rangle ,\;s,t\in \{0,1,2\}.$$ (7)

The important unitary transformation in a three-level quantum system called a generalized CNOT (GCNOT) gate is defined as [51]:

$$GCNOT(|m\rangle ,|n\rangle )=|m\rangle |\vec{m\;+\;n}\rangle ,\;m,n\in \{0,1,2\}$$ (8)

and the inverse GCNOT (IGCNOT) gate is expressed as:

$$IGCNOT(|m\rangle ,|n\rangle )=|m\rangle |\overset{\leftarrow }{m-n}\rangle ,\;m,n\in \{0,1,2\},$$ (9)

here, the first qutrit acts as the control, and the second as the target. and the expressions ‘$\vec{m\;+\;n}$’ and ‘$\overset{\leftarrow }{m-n}$’ represent the addition operation of module 3 and subtraction of modulo 3, respectively.

An arbitrary single-qudit state in a *d*-dimensional quantum system can be written as $|\varphi \rangle =\sum_{j=0}^{d-1}\alpha_{j}|j\rangle$, where the real numbers $\alpha_{j}$, j=0,1,⋯,d−i, satisfy $\sum_{j=0}^{d-1}\alpha_{j}^{2}=1$, while $\theta_{0}=0$ and $\theta_{j}$ is arbitrary real number for any j∈{0,1,⋯,d−1}. The *d*-Bell states of *d*-dimensional system is represented as [52]

$$|{DB}_{mn}\rangle =\frac{1}{\sqrt{d}}\sum_{k=0}^{d-1}\exp\{\frac{2\pi i}{d}km\}|k\rangle |(k\;+\;n)modd\rangle ,$$ (10)

where m,n=0,1,⋯,d−1 index the ${\text{d}}^{2}$ orthogonal Bell stats. The computational basis $Z_{d}=\{|k\rangle {\}}_{k=0}^{d\;-\;1}$ is the standard orthonormal basis, and the states |0⟩,|1⟩,⋯, and |d−1⟩ are eigenvectors of *Z*_*d*_ . In addition to the computational basis, we can define the complementary Fourier basis $X_{d}=\{|r\rangle^{x}{\}}_{r=0}^{d\;-\;1}$,whose eigenvectors are given by:

$$|r\rangle^{x}=\frac{1}{\sqrt{d}}\sum_{k=0}^{d\;-\;1}\exp\{\frac{2\pi i}{d}kr\}|k\rangle ,$$ (11)

where r=0,1,⋯,d−1. These bases are related by the condition $|\langle k|r\rangle^{x}|=1/\sqrt{d}$,meaning they are unbiased.This formulation highlights the essential properties of the computational and Fourier bases, and their use in the construction of the Bell states, ensuring the fidelity of quantum information transmission.

In 2002, Karimipour and Bahraminasab [52] proposed a GCNOT gate tailored for *d*-dimensional quantum systems,given by:

DCNOT(|k⟩,|l⟩)=|k⟩|(k+l)modd⟩,  k,l∈{0,1,⋯,d−1} (12)

and the inverse DCNOT (IDCNOT) gate can be expressed as:

IDCNOT(|k⟩,|l⟩)=|k⟩|(k−l)modd⟩,  k,l∈{0,1,⋯,d−1}, (13)

where the first qudit and the second qudit are the control qudit and the target qudit respectively.This family of operations provides fundamental tools for the preparation of high-dimensional entangled states, and their modular arithmetic properties are directly related to the phase synchronization mechanism in cyclic cloning protocols.

In practical quantum systems, noise interference is inevitably present, with AD noise being a typical non-unitary disturbance source that can effectively model energy dissipation, spontaneous photon emission, and other processes in quantum computing [52]. Under the Born-Markov approximation, the Kraus operators for single-qubit AD noise are represented as

$$K_{0}=\left(\begin{array}{cc} 1 & 0\\ 0 & \sqrt{1\;-\;\gamma } \end{array}\right),\;K_{1}=\left(\begin{array}{cc} 0 & \sqrt{\gamma }\\ 0 & 0 \end{array}\right),$$ (14)

where $\gamma =1\;-\;e^{-\tau t}\in [0,1]$ (τ>0) is the noise strength parameter. It is worth noting that the extension of this noise model to *d*-dimensional systems introduces more complex dissipation paths, posing special challenges for the fidelity analysis of high-dimensional cyclic cloning protocols. Similarly, the corresponding Kraus operators of AD noise about the single qutrit [54] can be expressed as:

$$F_{0}=\left(\begin{array}{ccc} 1 & 0 & 0\\ 0 & \sqrt{1\;-\;\gamma } & 0\\ 0 & 0 & \sqrt{1\;-\;\gamma } \end{array}\right),\;F_{1}=\left(\begin{array}{ccc} 0 & \sqrt{\gamma } & 0\\ 0 & 0 & 0\\ 0 & 0 & 0 \end{array}\right),\;F_{2}=\left(\begin{array}{ccc} 0 & 0 & \sqrt{\gamma }\\ 0 & 0 & 0\\ 0 & 0 & 0 \end{array}\right).$$ (15)

In Ref. [52], a high-dimensional generalization of AD noise was established, and the corresponding Kraus operators can be expressed as:

$$E_{0}=|0\rangle \langle 0|\;+\;\sqrt{1\;-\;\gamma }\sum_{j=1}^{d\;-\;1}|j\rangle \langle j|$$ (16)

and

$$E_{j}=\sqrt{\gamma }|0\rangle \langle j|\;(j=1,2,\cdots ,d\;-\;1)$$ (17)

This noise can be interpreted as follows: when a *d*-level quantum system is coupled to its environment, particles in the excited states |j⟩ (1≤j≤d−1) undergo non-radiative transitions with probability γ, leading to a collective relaxation process where the system transitions from the excited state levels to the ground state |0⟩. Specifically, the operator *E*_0_ represents the probability amplitude for the system to either remain in its original state or undergo partial dissipation, while *E*_*j*_ describes the complete de-excitation process from the *j*-th excited state to the ground state |0⟩.

## 3 Cyclic assisted cloning of arbitrary unknown single-qubit states in amplitude damping channel

In this section, we explore the conclusive results of cyclic assisted cloning in a two-dimensional quantum system, specifically focusing on the cloning of arbitrary unknown single-qubit states within the AD channel. To simplify our analysis, we assume that all qubits transmitted through the quantum channel experience the same independent AD strength. Meanwhile, the qubits that remain local are unaffected by this process. This setup allows us to isolate the effects of the AD channel on the transmitted qubits, while maintaining the integrity of those qubits that are kept in a controlled local environment.

### 3.1 Three-party cyclic assisted cloning in amplitude damping channel

In this scenario, four legitimate participants are considered: Alice, Bob, Charlie, and Victor. Victor is a state preparer who has prepared three pure quantum states, denoted as $|\xi \rangle_{A^{\prime }}$, $|\eta \rangle_{B^{\prime }}$, and $|\gamma \rangle_{C^{\prime }}$, for Alice, Bob, and Charlie, respectively. These states can be written as

$$|\xi \rangle_{A^{\prime }}=(\alpha |0\rangle \;+\;\beta |1\rangle {)}_{A^{\prime }},\;|\eta \rangle_{B^{\prime }}=(a|0\rangle \;+\;b|1\rangle {)}_{B^{\prime }},\;|\gamma \rangle_{C^{\prime }}=(x|0\rangle \;+\;y|1\rangle {)}_{C^{\prime }},$$ (18)

where α, *a*, and *x* are real numbers, while β, *b*, and *y* are complex numbers. These coefficients satisfy the normalization conditions $|\alpha {|}^{2}\;+\;|\beta {|}^{2}=1$, $|a{|}^{2}\;+\;|b{|}^{2}=1$, and $|x{|}^{2}\;+\;|y{|}^{2}=1$. Victor knows these coefficients, but they remain unknown to the other participants. Once Victor sends the particles $A^{\prime }$, $B^{\prime }$, and $C^{\prime }$ to Alice, Bob, and Charlie respectively, they each use the received states as their respective input states. Importantly, except for Victor, the other participants are unaware of the nature of these input states. The objective for Alice, Bob, and Charlie is to sequentially teleport their individual input states to the next participant in the sequence: Alice aims to teleport her state to Bob, Bob to Charlie, and Charlie to Alice. With Victor’s assistance, the participants aim to generate copies or OCC of their respective input states at their respective locations.To better illustrate the process, we have provided S1 Fig, which shows the relationships among the four legitimate participants—Alice, Bob, Charlie, and Victor. The diagram clearly depicts the quantum state interactions among the participants, making the overall workflow more intuitive and significantly enhancing the readability of the content. This scheme consists of three stages: preparation of quantum channels, cyclic quantum teleportation, and assisted cloning, with a total of ten steps, which are described as:

**Stage 1** Preparation of quantum channels.

(a1) To begin the process, Alice prepares a Bell state $|{ℬ}_{00}\rangle =\frac{1}{\sqrt{2}}(|00\rangle \;+\;|11\rangle {)}_{AA_{1}}$. She then sends qubit *A*_1_ to Bob through an AD channel.

(a2) Upon receiving qubit *A*_1_, Bob introduces an ancillary qubit *B* initialized to $|0\rangle_{B}$. He then performs a CNOT operation on the qubit pair (*A*_1_,*B*), with qubit *A*_1_ as the control and qubit *B* as the target. Afterward, Bob sends qubit *A*_1_ back to Alice through an AD channel.

(a3) Once Alice receives qubit *A*_1_, she applies another CNOT transformation on the qubit pair (*A*,*A*_1_), where qubit *A* serves as the control qubit and qubit *A*_1_ as the target. She then performs a single-qubit projective measurement on *A*_1_ in the computational basis {|0⟩,|1⟩}. If the outcome is $|0\rangle_{A_{1}}$, the process continues to Stage 2, step (b1); otherwise, the process is aborted and restarted until Alice obtains a successful outcome of $|0\rangle_{A_{1}}$.

It is important to note that, when Alice successfully obtains the outcome $|0\rangle_{A_{1}}$ in step (a3), the two qubits (*A*,*B*) are transformed into a PES:

$$|ℋ\rangle_{AB}=\frac{1}{\sqrt{1\;+\;(1\;-\;\gamma {)}^{2}}}[|00\rangle \;+\;(1\;-\;\gamma )|11\rangle {]}_{AB},$$ (19)

which detailed proof is given in S1 Appendix . That is, Alice and Bob successfully share this PES.

Similar to the above approach, Bob and Charlie can successfully share the PES

$$|ℋ\rangle_{B_{1}C}=\frac{1}{\sqrt{1\;+\;(1\;-\;\gamma {)}^{2}}}[|00\rangle \;+\;(1\;-\;\gamma )|11\rangle {]}_{B_{1}C},$$ (20)

and Charlie and Alice can also successfully share the PES

$$|ℋ\rangle_{C_{1}A_{2}}=\frac{1}{\sqrt{1\;+\;(1\;-\;\gamma {)}^{2}}}[|00\rangle \;+\;(1\;-\;\gamma )|11\rangle {]}_{C_{1}A_{2}}.$$ (21)

Therefore, the composite system of the states $|ℋ\rangle_{AB}$, $|ℋ\rangle_{B_{1}C}$ and $|ℋ\rangle_{C_{1}A_{2}}$ as well as the input states $|\xi \rangle_{A}$, $|\eta \rangle_{B}$ and $|\gamma \rangle_{C}$ is

$$|𝒢\rangle =|\xi \rangle_{A^{\prime }}⊗|\eta \rangle_{B^{\prime }}⊗|\gamma \rangle_{C^{\prime }}⊗|ℋ\rangle_{AB}⊗|ℋ\rangle_{B_{1}C}⊗|ℋ\rangle_{C_{1}A_{2}}.$$ (22)

**Stage 2** Cyclic quantum teleportation (CQT).

(b1)Alice and Bob each perform a Bell state measurement on the particle pairs $\left(A,A^{\prime }\right)$ and $\left(B_{1},B^{\prime }\right)$, respectively. Meanwhile, Charlie carries out a Bell state measurement on the particle pair $\left(C_{1},C^{\prime }\right)$. After completing these measurements, Alice, Bob, and Charlie share their results through classical communication channels. Using these Bell states, the state |𝒢⟩ of the composite system can be rewritten as

$$|𝒢\rangle =\frac{1}{\sqrt{[2\;+\;2(1\;-\;\gamma {)}^{2}{]}^{3}}}\{\sum_{s,t=0}^{1}|{ℬ}_{st}\rangle_{AA^{\prime }}[\alpha^{s⊕t⊕1}\beta^{s⊕t}|0\rangle \;+\;(-1{)}^{s}(1\;-\;\gamma )\alpha^{s⊕t}\beta^{s⊕t⊕1}|1\rangle {]}_{B}\}\;\;⊗\{\sum_{m,n=0}^{1}|{ℬ}_{mn}\rangle_{B_{1}B^{\prime }}[a^{m⊕n⊕1}b^{m⊕n}|0\rangle \;+\;(-1{)}^{m}(1\;-\;\gamma )a^{m⊕n}b^{m⊕n⊕1}|1\rangle {]}_{C}\}\;\;⊗\{\sum_{j,k=0}^{1}|{ℬ}_{jk}\rangle_{C_{1}C^{\prime }}[x^{j⊕k⊕1}y^{j⊕k}|0\rangle \;+\;(-1{)}^{j}(1\;-\;\gamma )x^{j⊕k}y^{j⊕k⊕1}|1\rangle {]}_{A_{2}}\}.$$ (23)

From Eq (23) it is straightforward to calculate the probability of Alice’s outcome $|{ℬ}_{st}\rangle_{AA^{\prime }}$, which is $\left[|\alpha^{s⊕t⊕1}\beta^{s⊕t}{|}^{2}\;+\;{\left(1\;-\;\gamma \right)}^{2}|\alpha^{s⊕t}\beta^{s⊕t⊕1}{|}^{2}]/2[1\;+\;{\left(1\;-\;\gamma \right)}^{2}\right]$. The probability of Bob or Charlie’s measurement is similar. In general, if the measurement results for Alice, Bob, and Charlie are $|{ℬ}_{st}\rangle_{AA^{\prime }}$, $|{ℬ}_{mn}\rangle_{B_{1}B^{\prime }}$ and $|{ℬ}_{jk}\rangle_{C_{1}C^{\prime }}$, respectively. Then the state of the qubits *B*, *C* and *A*_2_ collapses into

$$|{𝒢}^{\prime }\rangle =[\alpha^{s⊕t⊕1}\beta^{s⊕t}|0\rangle \;+\;(-1{)}^{s}(1\;-\;\gamma )\alpha^{s⊕t}\beta^{s⊕t⊕1}|1\rangle {]}_{B}\;\;⊗[a^{m⊕n⊕1}b^{m⊕n}|0\rangle \;+\;(-1{)}^{m}(1\;-\;\gamma )a^{m⊕n}b^{m⊕n⊕1}|1\rangle {]}_{C}\;\;⊗[x^{j⊕k⊕1}y^{j⊕k}|0\rangle \;+\;(-1{)}^{j}(1\;-\;\gamma )x^{j⊕k}y^{j⊕k⊕1}|1\rangle {]}_{A_{2}}.\;$$ (24)

(b2) According to measurement information, Alice, Bob and Charlie perform Pauli gates $\sigma^{\left(j,j\right)}$, $\sigma^{\left(m,m\right)}$ and $\sigma^{\left(s,s\right)}$ on *A*_2_, *B* and *C*, respectively. Therefore, the state $|{𝒢}^{\prime }\rangle$ as represented in Eq (24), is modified to

$$|{𝒢}^{\prime \prime }\rangle =[\alpha^{s⊕t⊕1}\beta^{s⊕t}|0\rangle \;+\;(1\;-\;\gamma )\alpha^{s⊕t}\beta^{s⊕t⊕1}|1\rangle {]}_{B}\;\;⊗[a^{m⊕n⊕1}b^{m⊕n}|0\rangle \;+\;(1\;-\;\gamma )a^{m⊕n}b^{m⊕n⊕1}|1\rangle {]}_{C}\;\;⊗[x^{j⊕k⊕1}y^{j⊕k}|0\rangle \;+\;(1\;-\;\gamma )x^{j⊕k}y^{j⊕k⊕1}|1\rangle {]}_{A_{2}}.\;$$ (25)

(b3) Bob prepares an auxiliary qubit *B*_2_ initialized in the state $|0\rangle_{B_{2}}$, and subsequently applies a unitary operation *U*_*B*_ on the joint system comprising qubits *B* and *B*_2_. In the computational basis $\left\{|00\rangle_{BB_{2}},|01\rangle_{BB_{2}},|10\rangle_{BB_{2}},|00\rangle_{BB_{2}}\right\}$, the operation *U*_*B*_ can be represented by the following 4×4 unitary matrix:

$$U_{B}=\left(\begin{array}{cccc} 1\;-\;\gamma & \sqrt{1-(1\;-\;\gamma {)}^{2}} & 0 & 0\\ \sqrt{1-(1\;-\;\gamma {)}^{2}} & \gamma -1 & 1 & 1\\ 0 & 0 & 1 & 0\\ 0 & 0 & 0 & 0 \end{array}\right).$$ (26)

After Bob’s unitary transformation *U*_*B*_, the state $[\alpha^{s⊕t⊕1}\beta^{s⊕t}|0\rangle \;+\;(1\;-\;\gamma )\alpha^{s⊕t}\beta^{s⊕t⊕1}|1\rangle {]}_{B}|0\rangle_{B_{2}}$ of qubits *B* and *B*_2_ is transformed into

$$U_{B}\{\frac{1}{\sqrt{1\;+\;(1\;-\;\gamma {)}^{2}}}[\alpha^{s⊕t⊕1}\beta^{s⊕t}|0\rangle \;+\;(1\;-\;\gamma )\alpha^{s⊕t}\beta^{s⊕t⊕1}|1\rangle {]}_{B}|0\rangle_{B_{2}}\}\;=\frac{1\;-\;\gamma }{\sqrt{1\;+\;(1\;-\;\gamma {)}^{2}}}[\alpha^{s⊕t⊕1}\beta^{s⊕t}|0\rangle \;+\;\alpha^{s⊕t}\beta^{s⊕t⊕1}|1\rangle {]}_{B}|0\rangle_{B_{2}}\;\;\;+\;\frac{\sqrt{1-(1\;-\;\gamma {)}^{2}}}{\sqrt{1\;+\;(1\;-\;\gamma {)}^{2}}}\alpha^{s⊕t⊕1}\beta^{s⊕t}|0\rangle_{B}|1\rangle_{B_{2}}.$$ (27)

Subsequently, Bob measures the auxiliary qubit *B*_2_ in *Z*-basis. If his measurement result is $|0\rangle_{B_{2}}$, Bob gets the state $[\alpha^{s⊕t⊕1}\beta^{s⊕t}|0\rangle \;+\;\alpha^{s⊕t}\beta^{s⊕t⊕1}|1\rangle {]}_{B}$, and then Bob applies Pauli gate $\sigma^{\left(0,s⊕t\right)}$ to his qubit *B*:


$$\sigma^{\left(0,s⊕t\right)}[\alpha^{s⊕t⊕1}\beta^{s⊕t}|0\rangle \;+\;\alpha^{s⊕t}\beta^{s⊕t⊕1}|1\rangle {]}_{B}=(\alpha |0\rangle \;+\;\beta |1\rangle {)}_{B},$$

which means that Bob successfully recovers Alice’s original state $|\xi \rangle_{A^{\prime }}$ on his qubit *B* when the outcome of the auxiliary qubit *B*_2_ is |0⟩. However, if the measurement result of *B*_2_ is |1⟩, the scheme fails.

According to Eq (27), the probability that Bob obtains the result $|0\rangle_{B_{2}}$ is given by ${\left(1\;-\;\gamma \right)}^{2}/[|\alpha^{s⊕t⊕1}\beta^{s⊕t}{|}^{2}\;+\;{\left(1\;-\;\gamma \right)}^{2}|\alpha^{s⊕t}\beta^{s⊕t⊕1}{|}^{2}]$. Form the above derivation, assuming Alice’s measurement result is $|{ℬ}_{st}\rangle_{AA^{\prime }}$, it follows that the probability for Bob to successfully reconstruct Alice’s original state equals ${\left(1\;-\;\gamma \right)}^{2}/[|\alpha^{s⊕t⊕1}\beta^{s⊕t}{|}^{2}\;+\;{\left(1\;-\;\gamma \right)}^{2}|\alpha^{s⊕t}\beta^{s⊕t⊕1}{|}^{2}]\;\times \;[|\alpha^{s⊕t⊕1}$ $\beta^{s⊕t}{|}^{2}\;+\;{\left(1\;-\;\gamma \right)}^{2}|\alpha^{s⊕t}\beta^{s⊕t⊕1}{|}^{2}]/2[1\;+\;{\left(1\;-\;\gamma \right)}^{2}]={\left(1\;-\;\gamma \right)}^{2}/2[1\;+\;{\left(1\;-\;\gamma \right)}^{2}]$.

Similar to what Bob did in (b3),as indicated by Eq (25) that Charlie can recover Bob’s state $|\eta \rangle_{B^{\prime }}$ on qubit *C* with probability ${\left(1\;-\;\gamma \right)}^{2}/2[1\;+\;{\left(1\;-\;\gamma \right)}^{2}]$, and Alice can also reconstruct Charlie’s state $|\gamma \rangle_{C^{\prime }}$ on her qubit *A*_2_ with probability ${\left(1\;-\;\gamma \right)}^{2}/2[1\;+\;{\left(1\;-\;\gamma \right)}^{2}]$. Therefore, the probability that Alice, Bob, and Charlie can successfully reconstruct the original states |γ⟩, |ξ⟩ and |η⟩ when the measurement results $|{ℬ}_{st}\rangle_{AA^{\prime }}$, $|{ℬ}_{mn}\rangle_{B_{1}B^{\prime }}$ and $|{ℬ}_{jk}\rangle_{C_{1}C^{\prime }}$ occur simultaneously is ${\left(1\;-\;\gamma \right)}^{6}/8[1\;+\;{\left(1\;-\;\gamma \right)}^{2}{]}^{3}$ i.e., for a fixed joint measurement outcome $|{ℬ}_{st}\rangle_{AA^{\prime }}|{ℬ}_{mn}\rangle_{B_{1}B^{\prime }}|{ℬ}_{jk}\rangle_{C_{1}C^{\prime }}$, the success probability of the cyclic QT is ${\left(1\;-\;\gamma \right)}^{6}/8[1\;+\;{\left(1\;-\;\gamma \right)}^{2}{]}^{3}$. From the former analysis, s,t,m,n,j,k∈{0,1}, that is, Alice, Bob and Charlie each have 4 measurement outcomes. It means that the joint measurement outcomes they constitute have a total of 64, so the total probability of our scheme is $8{\left(1\;-\;\gamma \right)}^{6}/[1\;+\;{\left(1\;-\;\gamma \right)}^{2}{]}^{3}$.

Note that when the noise strength of AD channel is zero (i.e., γ=0, there is no noise), then the states as shown in Eqs (19), (20), and (21) are all the same Bell state $|{ℬ}_{00}\rangle$, and the probability of our scheme is $8{\left(1\;-\;\gamma \right)}^{6}/[1\;+\;{\left(1\;-\;\gamma \right)}^{2}{]}^{3}=1$. In this case our scheme is the standard cyclic QT. In other words, the cyclic QT here is a generalization of the standard cyclic QT.

**Stage 3** Assisted cloning.

(c1) In the third phase of the protocol, if Alice, Bob, and Charlie each intend to obtain either a replica or an orthogonal complement of an unknown single-qubit state—denoted as $|\xi \rangle_{A^{\prime }}$, $|\eta \rangle_{B^{\prime }}$, and $|\gamma \rangle_{C^{\prime }}$, respectively—they must rely on the assistance of a state preparer, Victor. At first glance, this may appear infeasible due to the well-known no-cloning theorem in quantum mechanics, which forbids the duplication of arbitrary unknown quantum states. In particular, the application of Bell-state measurements could be thought to disturb the quantum correlations between the entangled pairs $\left(A,A^{\prime }\right)$, $\left(B_{1},B^{\prime }\right)$, and $\left(C_{1},C^{\prime }\right)$. However, from the perspective of quantum measurement theory, performing bipartite projective measurements—such as Bell-state projections—does not inherently violate the no-cloning principle. In physical implementations, such as those involving photons, Bell-state analysis typically constitutes a non-degenerate measurement, meaning that the process does not necessarily destroy the original quantum information [17].Therefore, the Bell-state measurements $|ℬst\rangle AA^{\prime }$, $|ℬmn\rangle B_{1}B^{\prime }$, and $|ℬjk\rangle C_{1}C^{\prime }$ can be performed without disturbing the original quantum states.

(c2) To accomplish the cloning operation, each of the three parties sends one particle from their entangled pair to Victor. Specifically, Alice sends particle $A^{\prime }$ to Victor while retaining particle *A*; Bob transfers $B^{\prime }$ to Victor and keeps *B*_1_; Charlie transmits $C^{\prime }$ to Victor and retains *C*_1_. A central question arises: given that the input states are unknown, can Victor assist the three parties in simultaneously reconstructing their respective original quantum states or their orthogonal complements? We will demonstrate that this is theoretically achievable. Suppose Alice, Bob, and Charlie each apply the projection operator $|{ℬ}_{00}\rangle \langle {ℬ}_{00}|$ on the entangled state |𝒢⟩ (see Eq (23)); then the six-particle system comprising $A,A^{\prime },B_{1},B^{\prime },C_{1},C^{\prime }$ collapses to the state $|{ℬ}_{00}\rangle_{AA^{\prime }}|{ℬ}_{00}\rangle_{B_{1}B^{\prime }}|{ℬ}_{00}\rangle_{C_{1}C^{\prime }}$, forming three pairs of perfect entanglement.

(c3) Since Victor has complete prior knowledge of the input states $|\xi \rangle_{A^{\prime }}$, $|\eta \rangle_{B^{\prime }}$, and $|\gamma \rangle_{C^{\prime }}$, he can perform a joint measurement on the three-particle system $\left(A^{\prime },B^{\prime },C^{\prime }\right)$ using an orthonormal and complete basis $\left\{|\varepsilon_{j}\rangle_{A^{\prime }B^{\prime }C^{\prime }}:j=1,\ldots ,8\right\}$. These measurement basis states are related to the computational basis of three qubits via the following transformation:

$$\left(\begin{array}{c} |\varepsilon_{1}\rangle \\ |\varepsilon_{2}\rangle \\ |\varepsilon_{3}\rangle \\ |\varepsilon_{4}\rangle \\ |\varepsilon_{5}\rangle \\ |\varepsilon_{6}\rangle \\ |\varepsilon_{7}\rangle \\ |\varepsilon_{8}\rangle \end{array}\right)=𝒲\left(\begin{array}{c} |000\rangle \\ |001\rangle \\ |010\rangle \\ |011\rangle \\ |100\rangle \\ |101\rangle \\ |110\rangle \\ |111\rangle \end{array}\right),$$ (28)

where

$$𝒲=\left(\begin{array}{cccccccc} \alpha ax & \alpha ay & \alpha bx & \alpha by & \beta ax & \beta ay & \beta bx & \beta by\\ -\alpha ay^{*} & \alpha ax & -\alpha by^{*} & \alpha bx & -\beta ay^{*} & \beta ax & -\beta by^{*} & \beta bx\\ -\alpha b^{*}x & -\alpha b^{*}y & \alpha ax & \alpha ay & -\beta b^{*}x & -\beta b^{*}y & \beta ax & \beta ay\\ \alpha b^{*}y^{*} & -\alpha b^{*}x & -\alpha ay^{*} & \alpha ax & \beta b^{*}y^{*} & -\beta b^{*}x & \beta ay^{*} & \beta ax\\ -\beta^{*}ax & -\beta^{*}ay & -\beta^{*}bx & -\beta^{*}by & \alpha ax & \alpha ay & \alpha bx & \alpha by\\ \beta^{*}ay^{*} & -\beta^{*}ax & \beta^{*}by^{*} & -\beta^{*}bx & -\alpha ay^{*} & \alpha ax & -\alpha by^{*} & \alpha bx\\ \beta^{*}b^{*}x & \beta^{*}b^{*}y & -\beta^{*}ax & -\beta^{*}ay & -\alpha b^{*}x & -\alpha b^{*}y & \alpha ax & \alpha ay\\ -\beta^{*}b^{*}y^{*} & \beta^{*}b^{*}x & \beta^{*}ay^{*} & -\beta^{*}ax & \alpha b^{*}y^{*} & -\alpha b^{*}x & -\alpha ay^{*} & \alpha ax \end{array}\right).$$ (29)

It is evident that the vectors defined in Eqs (28) and (29) constitute a complete and mutually orthogonal basis in the 8-dimensional Hilbert space. Once Victor performs a projective measurement on qubits $A^{\prime }$, $B^{\prime }$, and $C^{\prime }$, he communicates the result to Alice, Bob, and Charlie through classical channels.

Now writing the state $|{ℬ}_{00}\rangle_{AA^{\prime }}|{ℬ}_{00}\rangle_{B_{1}B^{\prime }}|{ℬ}_{00}\rangle_{C_{1}C^{\prime }}$ of qubits $A,A^{\prime },B_{1},B^{\prime },C_{1}$ and $C^{\prime }$ in the basis $\left\{|\varepsilon_{j}\rangle_{A^{\prime }B^{\prime }C^{\prime }}:j=1,2,\cdots ,8\right\}$ yields

$$\;|{ℬ}_{00}\rangle_{AA^{\prime }}|{ℬ}_{00}\rangle_{B_{1}B^{\prime }}|{ℬ}_{00}\rangle_{C_{1}C^{\prime }}\;=\frac{1}{2\sqrt{2}}[|\varepsilon_{1}\rangle_{A^{\prime }B^{\prime }C^{\prime }}(\alpha |0\rangle \;+\;\beta^{*}|1\rangle {)}_{A}(a|0\rangle \;+\;b^{*}|1\rangle {)}_{B_{1}}(x|0\rangle \;+\;y^{*}|1\rangle {)}_{C_{1}}\;\;\;+\;|\varepsilon_{2}\rangle_{A^{\prime }B^{\prime }C^{\prime }}(\alpha |0\rangle \;+\;\beta^{*}|1\rangle {)}_{A}(a|0\rangle \;+\;b^{*}|1\rangle {)}_{B_{1}}(x|1\rangle -y|0\rangle {)}_{C_{1}}\;\;\;+\;|\varepsilon_{3}\rangle_{A^{\prime }B^{\prime }C^{\prime }}(\alpha |0\rangle \;+\;\beta^{*}|1\rangle {)}_{A}(a|1\rangle -b|0\rangle {)}_{B_{1}}(x|0\rangle \;+\;y^{*}|1\rangle {)}_{C_{1}}\;\;\;+\;|\varepsilon_{4}\rangle_{A^{\prime }B^{\prime }C^{\prime }}(\alpha |0\rangle \;+\;\beta^{*}|1\rangle {)}_{A}(a|1\rangle -b|0\rangle {)}_{B_{1}}(x|1\rangle -y|0\rangle {)}_{C_{1}}\;\;\;+\;|\varepsilon_{5}\rangle_{A^{\prime }B^{\prime }C^{\prime }}(\alpha |1\rangle -\beta |0\rangle {)}_{A}(a|0\rangle \;+\;b^{*}|1\rangle {)}_{B_{1}}(x|0\rangle \;+\;y^{*}|1\rangle {)}_{C_{1}}\;\;\;+\;|\varepsilon_{6}\rangle_{A^{\prime }B^{\prime }C^{\prime }}(\alpha |1\rangle -\beta |0\rangle {)}_{A}(a|0\rangle \;+\;b^{*}|1\rangle {)}_{B_{1}}(x|1\rangle -y|0\rangle {)}_{C_{1}}\;\;\;+\;|\varepsilon_{7}\rangle_{A^{\prime }B^{\prime }C^{\prime }}(\alpha |1\rangle -\beta |0\rangle {)}_{A}(a|1\rangle -b|0\rangle {)}_{B_{1}}(x|0\rangle \;+\;y^{*}|1\rangle {)}_{C_{1}}\;\;\;+\;|\varepsilon_{8}\rangle_{A^{\prime }B^{\prime }C^{\prime }}(\alpha |1\rangle -\beta |0\rangle {)}_{A}(a|1\rangle -b|0\rangle {)}_{B_{1}}(x|1\rangle -y|0\rangle {)}_{C_{1}}.\;$$ (30)

According to Eq (30), it is clear that each measurement basis vector is associated with its corresponding collapsed state. This indicates that, upon receiving Victor’s measurement outcome, Alice, Bob, and Charlie can each infer the precise quantum state of their respective particle based on Victor’s announcement.

(c4)After receiving Victor’s measurement result, Alice, Bob, and Charlie can each apply suitable Pauli operations to recover their respective target quantum states. To better visualize this process, the entangled state $|{ℬ}_{00}\rangle_{AA^{\prime }}|{ℬ}_{00}\rangle_{B_{1}B^{\prime }}|{ℬ}_{00}\rangle_{C_{1}C^{\prime }}$ can be expanded as

$$\;|{ℬ}_{00}\rangle_{AA^{\prime }}|{ℬ}_{00}\rangle_{B_{1}B^{\prime }}|{ℬ}_{00}\rangle_{C_{1}C^{\prime }}\;=\frac{1}{2\sqrt{2}}[-|\varepsilon_{1}\rangle_{A^{\prime }BC^{\prime }}(i\sigma_{y}|\xi_{⟂}\rangle_{A})(i\sigma_{y}|\eta_{⟂}\rangle_{B_{1}})(i\sigma_{y}|\gamma_{⟂}\rangle_{C_{1}})\;\;\;+\;|\varepsilon_{2}\rangle_{A^{\prime }B^{\prime }C^{\prime }}(i\sigma_{y}|\xi_{⟂}\rangle_{A})(i\sigma_{y}|\eta_{⟂}\rangle_{B_{1}})(i\sigma_{y}|\gamma \rangle_{C_{1}})\;\;\;+\;|\varepsilon_{3}\rangle_{A^{\prime }B^{\prime }C^{\prime }}(i\sigma_{y}|\xi_{⟂}\rangle_{A})(i\sigma_{y}|\eta \rangle_{B_{1}})(i\sigma_{y}|\gamma_{⟂}\rangle_{C_{1}})\;\;-|\varepsilon_{4}\rangle_{A^{\prime }B^{\prime }C^{\prime }}(i\sigma_{y}|\xi_{⟂}\rangle_{A})(i\sigma_{y}|\eta \rangle_{B_{1}})(i\sigma_{y}|\gamma \rangle_{C_{1}})\;\;\;+\;|\varepsilon_{5}\rangle_{A^{\prime }B^{\prime }C^{\prime }}(i\sigma_{y}|\xi \rangle_{A})(i\sigma_{y}|\eta_{⟂}\rangle_{B_{1}})(i\sigma_{y}|\gamma_{⟂}\rangle_{C_{1}})\;\;-|\varepsilon_{6}\rangle_{A^{\prime }B^{\prime }C^{\prime }}(i\sigma_{y}|\xi \rangle_{A})(i\sigma_{y}|\eta_{⟂}\rangle_{B_{1}})(i\sigma_{y}|\gamma \rangle_{C_{1}})\;\;-|\varepsilon_{7}\rangle_{A^{\prime }B^{\prime }C^{\prime }}(i\sigma_{y}|\xi \rangle_{A})(i\sigma_{y}|\eta \rangle_{B_{1}})(i\sigma_{y}|\gamma_{⟂}\rangle_{C_{1}})\;\;\;+\;|\varepsilon_{8}\rangle_{A^{\prime }B^{\prime }C^{\prime }}(i\sigma_{y}|\xi \rangle_{A})(i\sigma_{y}|\eta \rangle_{B_{1}})(i\sigma_{y}|\gamma \rangle_{C_{1}}).\;$$ (31)

From Eq (31), it is apparent that Victor’s projective measurement on qubits $A^{\prime }$, $B^{\prime }$, and $C^{\prime }$ causes the remaining system, comprising *A*, *B*_1_, and *C*_1_, to collapse into a product state. Each of these resulting states corresponds to a single-qubit clone of the original input or its orthogonal complement, modified by the rotation $\sigma^{\left(1,0\right)}=i\sigma_{y}$.

Remarkably, irrespective of the measurement outcome, if Alice, Bob, and Charlie each apply the same local operation $i\sigma_{y}$ to their qubits, they can individually and concurrently obtain clones of either their original state or its orthogonal complement. Specifically: If Victor measures $|\varepsilon_{1}\rangle_{A^{\prime }B^{\prime }C^{\prime }}$, all three parties receive copies of their respective orthogonal complement states. For outcome $|\varepsilon_{2}\rangle_{A^{\prime }B^{\prime }C^{\prime }}$, Alice and Bob obtain orthogonal complements, while Charlie recovers his original state. If the result is $|\varepsilon_{3}\rangle_{A^{\prime }B^{\prime }C^{\prime }}$, Alice and Charlie receive orthogonal complements, and Bob retrieves his initial state. In the case of $|\varepsilon_{4}\rangle_{A^{\prime }B^{\prime }C^{\prime }}$, Bob and Charlie obtain their original states, while Alice gets her orthogonal complement. With $|\varepsilon_{5}\rangle_{A^{\prime }B^{\prime }C^{\prime }}$, Alice receives her original state, while Bob and Charlie obtain orthogonal complements. If the measurement yields $|\varepsilon_{6}\rangle_{A^{\prime }B^{\prime }C^{\prime }}$, Alice and Charlie reconstruct their original states, and Bob retrieves his orthogonal complement. For $|\varepsilon_{7}\rangle_{A^{\prime }B^{\prime }C^{\prime }}$, Alice and Bob obtain their original states, and Charlie gets the orthogonal complement. Finally, if the outcome is $|\varepsilon_{8}\rangle_{A^{\prime }B^{\prime }C^{\prime }}$, all three parties retrieve exact copies of their original unknown quantum states.

Extending this analysis to other 63 product Bell states listed in Eq (23), a similar conclusion holds. Regardless of which product Bell basis is observed, the same procedure allows each party to acquire either an exact replica or the orthogonal counterpart of their target state with perfect fidelity.

**Remark**: The 8×8 transformation matrix in Eq (29) can be decomposed as the tensor product of three 2×2 matrices

$$\left(\begin{array}{cc} \alpha & \beta \\ -\beta^{*} & \alpha \end{array}\right),\;\left(\begin{array}{cc} a & b\\ -b^{*} & a \end{array}\right),\;\left(\begin{array}{cc} x & y\\ -y^{*} & x \end{array}\right).$$ (32)

This implies that Victor utilizes the basis $|\varepsilon_{j}\rangle_{A^{\prime }B^{\prime }C^{\prime }}:j=1,2,\cdots ,8$ for simultaneous measurements on qubits $A^{\prime }$, $B^{\prime }$, and $C^{\prime }$. This is equivalent to measuring each qubit separately with the bases $\alpha |0\rangle \;+\;\beta |1\rangle ,\alpha |1\rangle -\beta^{*}|0\rangle$ for $A^{\prime }$, $a|0\rangle \;+\;b|1\rangle ,a|1\rangle -b^{*}|0\rangle$ for $B^{\prime }$, and $x|0\rangle \;+\;y|1\rangle ,x|1\rangle -y^{*}|0\rangle$ for $C^{\prime }$.

### 3.2 Multiparty cyclic assisted cloning of arbitrary unknown single-qubit states in amplitude damping channel

The previous section presented a three-party cyclic-assisted cloning protocol, supported by a designated state preparer. In this section, we extend the framework to a generalized scenario involving an *N*-party loop. Suppose there are N+1 authorized participants distributed across different spatial locations: Victor, Bob, and *Alice*_1_ through *Alice*_*N*_. As the state preparer, Victor generates *N* arbitrary single-qubit states given by

$$|\xi^{\left(j\right)}\rangle_{A_{j}}=\alpha_{j}|0\rangle_{A_{j}}\;+\;\beta_{j}|1\rangle_{A_{j}},$$ (33)

where j∈1,2,⋯,N, with each $\alpha_{j}$ being a real number and $\beta_{j}$ a complex number satisfying the normalization condition $|\alpha_{j}{|}^{2}\;+\;|\beta_{j}{|}^{2}=1$. These coefficients are completely known to Victor but entirely hidden from the remaining participants. Victor then distributes each qubit *A*_*j*_ to the corresponding Alice_*j*_, who treats it as her input state. Thus, apart from Victor, all other parties handle quantum states whose details are unknown to them. Each Alice_*j*_ aims to obtain either a faithful clone or an OCC of her input state, with Victor’s assistance. S2 Fig illustrates the cyclic assisted cloning relationship established among *N* Alices with the assistance of the quantum state preparer, Victor. The diagram clearly presents the complete workflow, significantly enhancing the readability and comprehensibility of the content.

Similar to Stage 1 of Sect 3.1, Alice_*j*_ shares a pure entangled state $|ℋ\rangle_{A_{j}^{\prime }A_{j\;+\;1}^{\prime \prime }}=\frac{1}{\sqrt{1\;+\;{\left(1\;-\;\gamma \right)}^{2}}}[|00\rangle \;+\;(1\;-\;\gamma )|11\rangle {]}_{A_{j}^{\prime }A_{j\;+\;1}^{\prime \prime }}$ with ${\text{Alice}}_{j\;+\;1}$, j=1,2,⋯,N, where N+1:≡1 and $A_{N\;+\;1}^{\prime \prime }:\equiv A_{1}^{\prime \prime }$. The composite system of the states $|ℋ\rangle_{A_{j}^{\prime }A_{j\;+\;1}^{\prime \prime }}$, and the input states $|\xi^{\left(j\right)}\rangle_{A_{j}}$ (j=1,2,⋯,N) can be written as

$$|\dot{G}\rangle ={⊗}_{j=1}^{N}|\xi^{\left(j\right)}\rangle_{A_{j}}{⊗}_{j=1}^{N}|ℋ\rangle_{A_{j}^{\prime }A_{j\;+\;1}^{\prime \prime }}.$$ (34)

The protocol proceeds in two main phases: cyclic quantum teleportation and assisted cloning. In the first phase, each Alice_*j*_ performs a sequence of operations:

**Step 1** Each Alice_*j*_ (j=1,2,⋯,N) performs a Bell-state measurement on her local qubit pair $\left(A_{j},A_{j}^{\prime }\right)$.This measurement projects the global system into a new entangled configuration, and as a result, the overall system collapses into a corresponding post-measurement state

$$|\dot{G}\rangle =\frac{1}{\sqrt{[2\;+\;2(1\;-\;\gamma {)}^{2}{]}^{N}}}{⊗}_{j=1}^{N}\{\sum_{s_{j},t_{j}=0}^{1}|{ℬ}_{s_{j}t_{j}}\rangle_{A_{j}^{\prime }A_{j}}[\alpha_{j}^{s_{j}⊕t_{j}⊕1}\beta_{j}^{s_{j}⊕t_{j}}|0\rangle \;\;\;+\;(-1{)}^{s_{j}}(1\;-\;\gamma )\alpha_{j}^{s_{j}⊕t_{j}}\beta_{j}^{s_{j}⊕t_{j}⊕1}|1\rangle {]}_{A_{j\;+\;1}^{\prime \prime }}\},$$ (35)

where $s_{j},t_{j}\in \{0,1\}$. From Eq (35) that the probability of Alice_*j*_’s measurement result $|{ℬ}_{s_{j}t_{j}}\rangle_{A_{j}^{\prime }A_{j}}$ is $\left[|\alpha_{j}^{s_{j}⊕t_{j}⊕1}\beta_{j}^{s_{j}⊕t_{j}}{|}^{2}\;+\;{\left(1\;-\;\gamma \right)}^{2}|\alpha_{j}^{s_{j}⊕t_{j}}\beta_{j}^{s_{j}⊕t_{j}⊕1}{|}^{2}]/2[1\;+\;{\left(1\;-\;\gamma \right)}^{2}\right]$. After completing their respective measurements, each Alice*j* broadcasts her result via classical communication channels. Suppose, in general, that Alice*j* obtains the Bell state $|ℬs_{j}t_{j}\rangle A_{j}^{\prime }A_{j}$ as her outcome for every j∈1,2,⋯,N. Under the influence of these measurement outcomes,the collapsed state of the remaining particles is

$$|\ddot{G}\rangle =\frac{1}{\sqrt{[2\;+\;2(1\;-\;\gamma {)}^{2}{]}^{N}}}{⊗}_{j=1}^{N}[\alpha_{j}^{s_{j}⊕t_{j}⊕1}\beta_{j}^{s_{j}⊕t_{j}}|0\rangle \;\;\;+\;(-1{)}^{s_{j}}(1\;-\;\gamma )\alpha_{j}^{s_{j}⊕t_{j}}\beta_{j}^{s_{j}⊕t_{j}⊕1}|1\rangle {]}_{A_{j\;+\;1}^{\prime \prime }}.$$ (36)

**Step 2** According to the Alice_*j*_’s measurement outcome, ${\text{Alice}}_{j\;+\;1}$ (j=1,2,⋯,N) performs the Pauli operation $\sigma^{\left(s_{j},s_{j}\right)}$ (*s*_*j*_ = 0,1) on her qubit $A_{j\;+\;1}^{\prime \prime }$, which changes the state $|\ddot{G}\rangle$ into

$$|{\ddot{G}}^{\prime }\rangle ={⊗}_{j=1}^{N}[\alpha_{j}^{s_{j}⊕t_{j}⊕1}\beta_{j}^{s_{j}⊕t_{j}}|0\rangle \;+\;(1\;-\;\gamma )\alpha_{j}^{s_{j}⊕t_{j}}\beta_{j}^{s_{j}⊕t_{j}⊕1}|1\rangle {]}_{A_{j\;+\;1}^{\prime \prime }}.$$ (37)

**Step 3** For each Alice_*j*_ (j=1,2,⋯,N), an auxiliary qubit ${\tilde{A}}_{j}$ is introduced in the initial state $|0\rangle_{{\tilde{A}}_{j}}$, followed by the application of the unitary operation $U_{{\tilde{A}}_{j}}$ to the qubits $A_{j\;+\;1}^{\prime \prime }$ and ${\tilde{A}}_{j}$. Under the basis $\{|00\rangle_{A_{j\;+\;1}^{\prime \prime }{\tilde{A}}_{j}}$, $|01\rangle_{A_{j\;+\;1}^{\prime \prime }{\tilde{A}}_{j}}$, $|10\rangle_{A_{j\;+\;1}^{\prime \prime }{\tilde{A}}_{j}}$, $|00\rangle_{A_{j\;+\;1}^{\prime \prime }{\tilde{A}}_{j}}\}$, the unitary transformation $U_{{\tilde{A}}_{j}}$ can be expressed as the following 4×4 matrix:

$$U_{{\tilde{A}}_{j}}=\left(\begin{array}{cccc} 1\;-\;\gamma & \sqrt{1-(1\;-\;\gamma {)}^{2}} & 0 & 0\\ \sqrt{1-(1\;-\;\gamma {)}^{2}} & \gamma -1 & 1 & 1\\ 0 & 0 & 1 & 0\\ 0 & 0 & 0 & 0 \end{array}\right).$$ (38)

After Alice_*j*_’s unitary transformation $U_{{\tilde{A}}_{j}}$, the state $[\alpha_{j}^{s_{j}⊕t_{j}⊕1}\beta_{j}^{s_{j}⊕t_{j}}|0\rangle \;+\;(1\;-\;\gamma )\alpha_{j}^{s_{j}⊕t_{j}}\beta_{j}^{s_{j}⊕t_{j}⊕1}|1\rangle {]}_{A_{j\;+\;1}^{\prime \prime }}|0\rangle_{{\tilde{A}}_{j}}$ of qubits $A_{j\;+\;1}^{\prime \prime }$ and ${\tilde{A}}_{j}$ is transformed into

$$U_{{\tilde{A}}_{j}}\{\frac{1}{\sqrt{1\;+\;(1\;-\;\gamma {)}^{2}}}[\alpha_{j}^{s_{j}⊕t_{j}⊕1}\beta_{j}^{s_{j}⊕t_{j}}|0\rangle \;+\;(1\;-\;\gamma )\alpha_{j}^{s_{j}⊕t_{j}}\beta_{j}^{s_{j}⊕t_{j}⊕1}|1\rangle {]}_{A_{j\;+\;1}^{\prime \prime }}|0\rangle_{{\tilde{A}}_{j}}\}\;=\frac{1\;-\;\gamma }{\sqrt{1\;+\;(1\;-\;\gamma {)}^{2}}}[\alpha_{j}^{s_{j}⊕t_{j}⊕1}\beta_{j}^{s_{j}⊕t_{j}}|0\rangle \;+\;\alpha_{j}^{s⊕t}\beta_{j}^{s⊕t⊕1}|1\rangle {]}_{A_{j\;+\;1}^{\prime \prime }}|0\rangle_{{\tilde{A}}_{j}}\;\;\;+\;\frac{\sqrt{1-(1\;-\;\gamma {)}^{2}}}{\sqrt{1\;+\;(1\;-\;\gamma {)}^{2}}}\alpha_{j}^{s_{j}⊕t_{j}⊕1}\beta_{j}^{s_{j}⊕t_{j}}|0\rangle_{A_{j\;+\;1}^{\prime \prime }}|1\rangle_{{\tilde{A}}_{j}}.$$ (39)

After that, Alice_*j*_ measures the auxiliary qubit ${\tilde{A}}_{j}$ in the computational basis. If the outcome of his measurement is $|0\rangle_{{\tilde{A}}_{j}}$, Alice_*j*_ obtains the state $[\alpha_{j}^{s_{j}⊕t_{j}⊕1}\beta_{j}^{s_{j}⊕t_{j}}|0\rangle \;+\;\alpha_{j}^{s_{j}⊕t_{j}}\beta_{j}^{s_{j}⊕t_{j}⊕1}|1\rangle {]}_{A_{j\;+\;1}^{\prime \prime }}$, and then Alice_*j*_ perform Pauli gate $\sigma^{\left(0,s_{j}⊕t_{j}\right)}$ to his qubit $A_{j\;+\;1}^{\prime \prime }$:


$$\sigma^{\left(0,s_{j}⊕t_{j}\right)}[\alpha_{j}^{s_{j}⊕t_{j}⊕1}\beta_{j}^{s_{j}⊕t_{j}}|0\rangle \;+\;\alpha_{j}^{s_{j}⊕t_{j}}\beta_{j}^{s_{j}⊕t_{j}⊕1}|1\rangle {]}_{A_{j\;+\;1}^{\prime \prime }}=(\alpha_{j}|0\rangle \;+\;\beta_{j}|1\rangle {)}_{A_{j\;+\;1}^{\prime \prime }},$$

which means that Alice_*j*_ has already successfully reconstructed the Alice_*j*−1_’s original state $|\xi^{\left(j-1\right)}\rangle_{A_{j-1}}$ on her qubit $A_{j\;+\;1}^{\prime \prime }$, where j=1,2,⋯,N and 0=1−1:≡N. While Alice_*j*_’s result is $|1\rangle_{{\tilde{A}}_{j}}$, the scheme is failed.

After all the Alices have performed the above series of operations, we have

$${⊗}_{j=1}^{N}U_{{\tilde{A}}_{j}}|{\ddot{G}}^{\prime }\rangle ={⊗}_{j=1}^{N}\{U_{{\tilde{A}}_{j}}[\alpha_{j}^{s_{j}⊕t_{j}⊕1}\beta_{j}^{s_{j}⊕t_{j}}|0\rangle \;+\;(1\;-\;\gamma )\alpha_{j}^{s_{j}⊕t_{j}}\beta_{j}^{s_{j}⊕t_{j}⊕1}|1\rangle {]}_{A_{j\;+\;1}^{\prime \prime }}\}\;={⊗}_{j=1}^{N}(\alpha_{j}|0\rangle \;+\;\beta_{j}|1\rangle {)}_{A_{j\;+\;1}^{\prime \prime }},$$ (40)

which means that for joint measurement result ${⊗}_{j=1}^{N}|{ℬ}_{s_{j}t_{j}}\rangle_{A_{j}^{\prime }A_{j}}$ come from Alice_1_, ALice_2_, ⋯, Alice_*N*_, cyclic quantum teleportation is completed.

According to Eq (39), we can compute the probability of Alice_*j*_ obtaining the result $|0\rangle_{{\tilde{A}}_{j}}$,which is given by ${\left(1\;-\;\gamma \right)}^{2}/[|\alpha_{j}^{s_{j}⊕t_{j}⊕1}\beta_{j}^{s_{j}⊕t_{j}}{|}^{2}\;+\;{\left(1\;-\;\gamma \right)}^{2}|\alpha_{j}^{s_{j}⊕t_{j}}\beta_{j}^{s_{j}⊕t_{j}⊕1}{|}^{2}]$. From this analysis, assuming Alice _*j*_’s result is $|{ℬ}_{s_{j}t_{j}}\rangle_{A_{j}^{\prime }A_{j}}$, it can be concluded that the success probability for Alice _*j*_ to reconstruct Alice _*j*−1_’s original state is ${\left(1\;-\;\gamma \right)}^{2}/[|\alpha_{j}^{s_{j}⊕t_{j}⊕1}\beta_{j}^{s_{j}⊕t_{j}}{|}^{2}\;+\;{\left(1\;-\;\gamma \right)}^{2}|\alpha_{j}^{s_{j}⊕t_{j}}$ $\beta_{j}^{s_{j}⊕t_{j}⊕1}{|}^{2}]\;\times \;[|\alpha_{j}^{s_{j}⊕t_{j}⊕1}\beta_{j}^{s_{j}⊕t_{j}}{|}^{2}\;+\;{\left(1\;-\;\gamma \right)}^{2}|\alpha_{j}^{s_{j}⊕t_{j}}\beta_{j}^{s_{j}⊕t_{j}⊕1}{|}^{2}]/2[1\;+\;{\left(1\;-\;\gamma \right)}^{2}]={\left(1\;-\;\gamma \right)}^{2}/2[1\;+\;{\left(1\;-\;\gamma \right)}^{2}]$. From the former analysis, $s_{j},t_{j}\in \{0,1\}$ and j∈{1,2,⋯,N}, so the total probability of our scheme is

$$p_{N}=\{2\;\times \;2\;\times \;\frac{(1\;-\;\gamma {)}^{2}}{2[1\;+\;(1\;-\;\gamma {)}^{2}]}{\}}^{N}=\frac{2^{N}(1\;-\;\gamma {)}^{2N}}{[1\;+\;(1\;-\;\gamma {)}^{2}{]}^{N}}.$$ (41)

Obviously, when γ=0, the cyclic QT scheme here is also a generalization of the standard cyclic QT scheme [36].

Now, we turn to the discussion of the last stage of our scheme, i.e., the assisted cloning stage. To create a copies for each of the unknown single-qubit states, all the Alices require assistance from the state preparer Victor. According to the projection postulate of quantum mechanics, if each Alice applies Bell state measurement onto the $|\dot{G}\rangle$ as shown in Eq (34), the state of qubit pairs $\left(A_{1}^{\prime },A_{1}\right)$, $\left(A_{2}^{\prime },A_{2}\right)$, ⋯, $\left(A_{N}^{\prime },A_{N}\right)$ will collapse into some product states of Bell states. Without loss of generality, we only consider the collapsed state ${⊗}_{j=1}^{N}|{ℬ}_{00}\rangle_{A_{j}^{\prime }A_{j}}$. Each Alice_*j*_ sends her qubit *A*_*j*_ to Victor and keeps qubit $A_{j}^{\prime }$ in her possession, where j=1,2,⋯,N.

To accomplish the assisted cloning task, we begin by defining the fundamental mutually orthogonal basis vectors *w*_*N*−1,*N*_ as follows:

$$w_{N-1,N}=\left(\begin{array}{cccc} \alpha_{N-1}\alpha_{N} & \alpha_{N-1}\beta_{N} & \beta_{N-1}\alpha_{N} & \beta_{N-1}\beta_{N}\\ -\alpha_{N-1}\beta_{N}^{*} & \alpha_{N-1}\alpha_{N} & -\beta_{N-1}\beta_{N}^{*} & \beta_{N-1}\alpha_{N}\\ -\beta_{N-1}^{*}\alpha_{N} & -\beta_{N-1}^{*}\beta_{N} & \alpha_{N-1}\alpha_{N} & \alpha_{N-1}\beta_{N}\\ \beta_{N-1}^{*}\beta_{N}^{*} & -\beta_{N-1}^{*}\alpha_{N} & -\alpha_{N-1}\beta_{N}^{*} & \alpha_{N-1}\alpha_{N} \end{array}\right),$$ (42)

and then the general basis vector *W*_*n*_ can be expressed as

$$W_{N}=\overset{N-2}{\underset{j=1}{⨂}}w_{j}⊗w_{N-1,N},$$ (43)

where

$$w_{j}=\left(\begin{array}{cc} \alpha_{j} & \beta_{j}\\ -\beta_{j}^{*} & \alpha_{j} \end{array}\right)\;(j=1,2,\cdots ,N-2).$$ (44)

Then, the set of mutually orthogonal basis vectors $\left\{|\varepsilon_{j}\rangle_{A_{1}A_{2}\cdots A_{N}}:j=1,2,\cdots ,2^{N}\right\}$ can be written as

$$(|\varepsilon_{1}\rangle ,|\varepsilon_{2}\rangle ,\cdots ,|\varepsilon_{2^{N}}\rangle {)}^{T}=W_{N}S^{T},$$ (45)

where S=(|00⋯00⟩,|00⋯01⟩,⋯,|11⋯11⟩) represents the standard orthogonal basis of the ${\text{d}}^{\text{N}}$-dimensional Hilbert space,and ${\text{S}}^{\text{T}}$ is its transpose.Accordingly, the state ${⊗}_{j=1}^{N}|{ℬ}_{00}\rangle_{A_{j}^{\prime }A_{j}}$ can be rewritten in the basis $\left\{|\varepsilon_{j}\rangle_{A_{1}A_{2}\cdots A_{N}}:j=1,2,\cdots ,2^{N}\right\}$ as

$$\;{⊗}_{j=1}^{N}|{ℬ}_{00}\rangle_{A_{j}^{\prime }A_{j}}\;=\frac{1}{\sqrt{2^{N}}}[|\varepsilon_{1}\rangle_{A_{1}A_{2}\cdots A_{N}}(\alpha_{1}|0\rangle \;+\;\beta_{1}^{*}|1\rangle {)}_{A_{1}^{\prime }}(\alpha_{2}|0\rangle \;+\;\beta_{2}^{*}|1\rangle {)}_{A_{2}^{\prime }}\cdots (\alpha_{N}|0\rangle \;+\;\beta_{N}^{*}|1\rangle {)}_{A_{N}^{\prime }}\;\;\;+\;|\varepsilon_{2}\rangle_{A_{1}A_{2}\cdots A_{N}}(\alpha_{1}|0\rangle \;+\;\beta_{1}^{*}|1\rangle {)}_{A_{1}^{\prime }}(\alpha_{2}|0\rangle \;+\;\beta_{2}^{*}|1\rangle {)}_{A_{2}^{\prime }}\cdots (\alpha_{N}|1\rangle -\beta_{N}|0\rangle {)}_{A_{N}^{\prime }}\;\;\;+\;\cdots \;\;\;+\;|\varepsilon_{2^{N}-1}\rangle_{A_{1}A_{2}\cdots A_{N}}(\alpha_{1}|1\rangle -\beta_{A_{1}^{\prime }}|0\rangle {)}_{1}(\alpha_{2}|1\rangle -\beta_{2}|0\rangle {)}_{A_{2}^{\prime }}\cdots (\alpha_{N}|0\rangle \;+\;\beta_{N}^{*}|1\rangle {)}_{A_{N}^{\prime }}\;\;\;+\;|\varepsilon_{2^{N}}\rangle_{A_{1}A_{2}\cdots A_{N}}(\alpha_{1}|1\rangle -\beta_{A_{1}^{\prime }}|0\rangle {)}_{1}(\alpha_{2}|1\rangle -\beta_{2}|1\rangle {)}_{A_{2}^{\prime }}\cdots (\alpha_{N}|1\rangle -\beta_{N}|0\rangle {)}_{A_{N}^{\prime }}.\;$$ (46)

Because Victor knows the quantum states as shown in Eq (33) completely, he is capable of performing a projective measurement on the qubits $A_{1},A_{2},\cdots ,A_{N}$ using a set of mutually orthogonal basis vectors denoted by $\left\{|\varepsilon_{j}\rangle_{A_{1}A_{2}\cdots A_{N}}:j=1,2,\cdots ,2^{N}\right\}$ . As evident from Eq (46), each basis vector corresponds uniquely to a specific collapsed quantum state. This implies that, once Victor announces his measurement outcome via classical communication, each participant Alice_*j*_ can independently determine the resulting state of her own qubit.

Based on Victor’s broadcasted outcome, every Alice_*j*_ can apply an appropriate Pauli operator to reconstruct her target state. To illustrate this process more clearly, we can rewrite the entangled state ${⊗}_{j=1}^{N}|{ℬ}_{00}\rangle_{A_{j}^{\prime }A_{j}}$ in the following form:

$$\;{⊗}_{j=1}^{N}|{ℬ}_{00}\rangle_{A_{j}^{\prime }A_{j}}\;=\frac{1}{\sqrt{2^{N}}}[(-1{)}^{N}|\varepsilon_{1}\rangle_{A_{1}A_{2}\cdots A_{N}}(i\sigma_{y}|\xi_{⟂}^{\left(1\right)}\rangle_{A_{1}^{\prime }})(i\sigma_{y}|\xi_{⟂}^{\left(2\right)}\rangle_{A_{2}^{\prime }})\cdots (i\sigma_{y}|\xi_{⟂}^{\left(N\right)}\rangle_{A_{N}^{\prime }})\;\;\;+\;(-1{)}^{N-1}|\varepsilon_{2}\rangle_{A_{1}A_{2}\cdots A_{N}}(i\sigma_{y}|\xi_{⟂}^{\left(1\right)}\rangle_{A_{1}^{\prime }})(i\sigma_{y}|\xi_{⟂}^{\left(2\right)}\rangle_{A_{2}^{\prime }})\cdots (i\sigma_{y}|\xi^{\left(N\right)}\rangle_{A_{N}^{\prime }})\;\;\;+\;\cdots \;\;-|\varepsilon_{2^{N}-1}\rangle_{A_{1}A_{2}\cdots A_{N}}(i\sigma_{y}|\xi^{\left(1\right)}\rangle_{A_{1}^{\prime }})(i\sigma_{y}|\xi^{\left(2\right)}\rangle_{A_{2}^{\prime }})\cdots (i\sigma_{y}|\xi_{⟂}^{\left(N\right)}\rangle_{A_{N}^{\prime }})\;\;\;+\;|\varepsilon_{2^{N}}\rangle_{A_{1}A_{2}\cdots A_{N}}(i\sigma_{y}|\xi^{\left(1\right)}\rangle_{A_{1}^{\prime }})(i\sigma_{y}|\xi^{\left(2\right)}\rangle_{A_{2}^{\prime }})\cdots (i\sigma_{y}|\xi^{\left(N\right)}\rangle_{A_{N}^{\prime }}).$$ (47)

As shown in Eq (47), once Victor projects the joint system of all qubit pairs $\left(A_{j}^{\prime },A_{j}\right)$ onto a specific measurement basis, the resulting state is a tensor product of the output states across qubits $A_{1}^{\prime },A_{2}^{\prime },\cdots ,A_{N}^{\prime }$, where each output corresponds to a transformed version—either a perfect clone or an orthogonal complement—of the original unknown state. Notably, regardless of which basis vector $|\varepsilon_{j}\rangle$ is observed, if all Alice_*j*_ parties apply the same Pauli operator $\sigma^{\left(1,0\right)}=i\sigma_{y}$ to their respective qubits, they can simultaneously generate the corresponding states. For example, if Victor’s measurement result is $|\varepsilon_{1}\rangle_{A_{1}A_{2}\cdots A_{N}}$, each Alice_*j*_ will obtain the OCC of her unknown input state. If the outcome is $|\varepsilon_{2}\rangle_{A_{1}A_{2}\cdots A_{N}}$, then the first *N*–1 Alices receive OCCs of their respective states, while the last Alice recovers an exact replica of her initial unknown state. This pattern continues: if Victor observes $|\varepsilon_{2^{N}-1}\rangle_{A_{1}A_{2}\cdots A_{N}}$, then the first *N*–1 Alices obtain perfect clones, while the final Alice gets the OCC; in the case of $|\varepsilon_{2^{N}}\rangle_{A_{1}A_{2}\cdots A_{N}}$, all Alices receive exact copies of their original unknown states.

Using an analogous approach, in the process of CCQT, if the measurement outcomes of all Alice_*j*_ correspond to any of the remaining $4^{\text{N}}$–1 product Bell states defined in Eq (35), then following the same reasoning, each Alice_*j*_ is still able to reconstruct either an exact replica or the orthogonal complement of her initial unknown single-qubit state.

## 4 Cyclic assisted cloning of arbitrary unknown single-qutrit states in amplitude damping channel

In this section, we present a multiparty cyclic-assisted cloning scheme for arbitrary unknown single-qutrit states in an AD channel, using a similar approach. We assume that the transmitted qutrits are affected by identical and independent AD noise, whereas the qutrits held locally remain unaffected by any noise.

We begin by focusing on a three-party cyclic-assisted cloning protocol involving four legitimate participants: Alice, Bob, Charlie, and Victor. In this setup, Victor acts as the state preparer and initially prepares three unknown pure qutrit states $|\xi \rangle_{A}$, $|\eta \rangle_{B}$ and $|\gamma \rangle_{C}$,as follows:

$$|\xi \rangle_{A}=(\alpha_{0}|0\rangle \;+\;\alpha_{1}e^{i\theta_{1}}|1\rangle \;+\;\alpha_{2}e^{i\theta_{2}}|2\rangle {)}_{A},\;|\eta \rangle_{B}=(\beta_{0}|0\rangle \;+\;\beta_{1}e^{i\vartheta_{1}}|1\rangle \;+\;\beta_{2}e^{i\vartheta_{2}}|2\rangle {)}_{B},\;|\zeta \rangle_{C}=(\lambda_{0}|0\rangle \;+\;\lambda_{1}e^{i\tau_{1}}|1\rangle \;+\;\lambda_{2}e^{i\tau_{2}}|2\rangle {)}_{C},$$ (48)

where $\alpha_{j}$, $\beta_{j}$ and $\lambda_{j}$ (j=0,1,2)are real numbers that satisfy the normalization conditions $\sum_{j=0}^{2}\alpha_{j}^{2}=1$, $\sum_{j=0}^{2}\beta_{j}^{2}=1$ and $\sum_{j=0}^{2}\lambda_{j}^{2}=1$.The phase parameters $\theta_{j}$, $\vartheta_{j}$, $\tau_{j}$ (j=1,2)are arbitrary real numbers. It is important to note that all of these parameters are known only to Victor and remain unknown to the other participants. After preparing the three qutrits, Victor sends qutrits *A*, *B* and *C* to Alice, Bob, and Charlie respectively. Each participant regards the received qutrit as their own input state. However, since the state parameters are hidden from them, they cannot determine the exact form of their respective quantum states. The primary objective of Alice, Bob, and Charlie is to teleport their unknown input states in a cyclic order—Alice to Bob, Bob to Charlie, and Charlie to Alice. With Victor’s assistance, they aim to generate either perfect clones or OCC of their original quantum states at their own locations.The schematic diagram of the cyclic assisted cloning scheme for arbitrary unknown single-qutrit states in the amplitude damping channel is very similar to that of the scheme in Sect 3. The main difference lies in the quantum states being transmitted. However, since the cyclic transmission method and Victor’s assistance mechanism remain unchanged, we will not add additional diagrammatic explanations here to ensure clarity and avoid redundancy. This scheme mainly includes three stages: preparation of quantum channels, cyclic quantum teleportation and assisted cloning, with a total of ten steps, which are described as follows:

**Stage 1** Preparation of quantum channels.

($\tilde{\text{a}}$1) To begin, Alice generates a Bell state $|{GB}_{00}\rangle =\frac{1}{\sqrt{3}}(|00\rangle \;+\;|11\rangle \;+\;|22\rangle {)}_{A^{\prime }A_{1}^{\prime }}$.She then transmits the qutrit $A_{1}^{\prime }$ to Bob through the AD channel.

($\tilde{\text{a}}$2) Upon reception of qutrit $A_{1}^{\prime }$, Bob initializes an ancillary qutrit *B*_1_ in the state $|0\rangle_{B_{1}}$. He applies a GCNOT operation on the qutrit pair $\left(A_{1}^{\prime },B_{1}\right)$, where $A_{1}^{\prime }$ acts as the control and *B*_1_ as the target. After completing this operation, Bob sends the qutrit $A_{1}^{\prime }$ back to Alice via the AD channel.

($\tilde{\text{a}}$3) Once Alice receives $A_{1}^{\prime }$, she first carries out IGCNOT transformation on qutrit pair $\left(A^{\prime },A_{1}^{\prime }\right)$, with $A^{\prime }$ acting as the control and $A_{1}^{\prime }$ as the target . Subsequently, she implements a single-qutrit projective measurement in computational basis {|0⟩,|1⟩,|2⟩} on qutrit $A_{1}^{\prime }$. If the measurement outcome is $|0\rangle_{A_{1}^{\prime }}$, then the scheme continues to ($\tilde{\text{b}}$1) in Stage 2; If not, the process restarts from the beginning until Alice successfully obtains the measurement outcome $|0\rangle_{A_{1}^{\prime }}$.

Noticing that in above ($\tilde{\text{a}}$3) when Alice gets the outcome $|0\rangle_{A_{1}^{\prime }}$ successfully, two qutrits $\left(A^{\prime },B_{1}\right)$ is transformed into a PES:

$$|ℋ\rangle_{A^{\prime }B_{1}}=\frac{1}{\sqrt{1\;+\;2(1\;-\;\gamma {)}^{2}}}[|00\rangle \;+\;(1\;-\;\gamma )|11\rangle \;+\;(1\;-\;\gamma )|22\rangle {]}_{A^{\prime }B_{1}},$$ (49)

which detailed proof is given in S2 Appendix. That is, Alice and Bob successfully share this PES.

Similar to the above approach, Bob and Charlie can successfully share the PES

$$|ℋ\rangle_{B^{\prime }C_{1}}=\frac{1}{\sqrt{1\;+\;2(1\;-\;\gamma {)}^{2}}}[|00\rangle \;+\;(1\;-\;\gamma )|11\rangle \;+\;(1\;-\;\gamma )|22\rangle {]}_{B^{\prime }C_{1}},$$ (50)

and Charlie and Alice can also successfully share the pure ent state

$$|ℋ\rangle_{C^{\prime }A_{1}}=\frac{1}{\sqrt{1\;+\;2(1\;-\;\gamma {)}^{2}}}[|00\rangle \;+\;(1\;-\;\gamma )|11\rangle \;+\;(1\;-\;\gamma )|22\rangle {]}_{C^{\prime }A_{1}}.$$ (51)

Therefore, the composite system consisting of the states $|ℋ\rangle_{A^{\prime }B_{1}}$, $|ℋ\rangle_{B^{\prime }C_{1}}$ and $|ℋ\rangle_{C^{\prime }A_{1}}$,along with the input states $|\xi \rangle_{A}$, $|\eta \rangle_{B}$ and $|\zeta \rangle_{C}$ is

$$|𝒬\rangle =|\xi \rangle_{A}⊗|\eta \rangle_{B}⊗|\zeta \rangle_{C}⊗|ℋ\rangle_{A^{\prime }B_{1}}⊗|ℋ\rangle_{B^{\prime }C_{1}}⊗|ℋ\rangle_{C^{\prime }A_{1}}.$$ (52)

**Stage 2** Circularly teleport quantum states

($\tilde{\text{b}}$1) Alice and Bob each perform measurements on their respective qutrit pairs $\left(A,A^{\prime }\right)$ and $\left(B,B^{\prime }\right)$ using the generalized Bell-state basis (as shown in Eq (7)). Simultaneously, Charlie conducts a generalized Bell-state measurement on the qutrit pair $\left(C,C^{\prime }\right)$. After completing the measurements, Alice, Bob, and Charlie each communicate their outcomes via classical channels, sending the results to Bob, Charlie, and Alice, respectively. Based on these generalized Bell-state projections, the overall state |𝒬⟩ of the composite system can be expressed as:

$$|𝒬\rangle =\frac{1}{\sqrt{[3\;+\;6(1\;-\;\gamma {)}^{2}{]}^{3}}}\;\;\;\times \;\{\sum_{s,t=0}^{2}|{GB}_{st}\rangle_{AA^{\prime }}\sum_{j=0}^{2}\alpha_{j}(1\;-\;\gamma {)}^{\min[1,(j\;+\;t)mod3]}e^{i(\theta_{j}-2\pi sj/3)}|(j\;+\;t)mod3\rangle_{B_{1}}\}\;\;⊗\{\sum_{m,n=0}^{2}|{GB}_{mn}\rangle_{BB^{\prime }}\sum_{l=0}^{2}\beta_{l}(1\;-\;\gamma {)}^{\min[1,(l\;+\;n)mod3]}e^{i(\vartheta_{l}-2\pi ml/3)}|(l\;+\;n)mod3\rangle_{C_{1}}\}\;\;⊗\{\sum_{u,v=0}^{2}|{GB}_{uv}\rangle_{CC^{\prime }}\sum_{k=0}^{2}\lambda_{k}(1\;-\;\gamma {)}^{\min[1,(k\;+\;v)mod3]}e^{i(\tau_{j}-2\pi uk/3)}|(k\;+\;v)mod3\rangle_{A_{1}}\},$$ (53)

where $\theta_{0}=0,\vartheta_{0}=0$ and $\tau_{0}=0$.

As observed from Eq (53), the probability that Alice obtains the outcome $|{GB}_{st}\rangle_{AA^{\prime }}$ can be written as $\left[\sum_{j=0}^{2}\alpha_{j}^{2}{\left(1\;-\;\gamma \right)}^{2\min[1,(j\;+\;t)mod3]}]/3[1\;+\;2{\left(1\;-\;\gamma \right)}^{2}\right]$. Bob and Charlie’s measurement probabilities take on a similar form. Once all the measurements are performed, Alice, Bob, and Charlie share their individual results through classical communication. For generality, suppose the outcomes are given by $|{GB}_{st}\rangle_{AA^{\prime }}$, $|{GB}_{mn}\rangle_{BB^{\prime }}$ and $|{GB}_{uv}\rangle_{CC^{\prime }}$, corresponding to Alice, Bob, and Charlie, respectively. Then the state of the qutrits *B*_1_, *C*_1_ and *A*_1_ collapses into

$$|{𝒬}^{\prime }\rangle =\{\sum_{j=0}^{2}\alpha_{j}(1\;-\;\gamma {)}^{\min[1,(j\;+\;t)mod3]}e^{i(\theta_{j}-2\pi sj/3)}|(j\;+\;t)mod3\rangle_{B_{1}}\}\;\;⊗\{\sum_{l=0}^{2}\beta_{l}(1\;-\;\gamma {)}^{\min[1,(l\;+\;n)mod3]}e^{i(\vartheta_{l}-2\pi ml/3)}|(l\;+\;n)mod3\rangle_{C_{1}}\}\;\;⊗\{\sum_{k=0}^{2}\lambda_{k}(1\;-\;\gamma {)}^{\min[1,(k\;+\;v)mod3]}e^{i(\tau_{j}-2\pi uk/3)}|(k\;+\;v)mod3\rangle_{A_{1}}\}.$$ (54)

($\tilde{\text{b}}$2) After hearing the measurement information, Alice, Bob and Charlie perform unitary operations *U*_*A*_, *U*_*b*_ and qutrits *U*_*C*_ on *A*_1_, *B*_1_ and *C*_1_, respectively, which are given by

$$U_{A}=\sum_{k=0}^{2}e^{2\pi iuk/3}|k\rangle \langle (k\;+\;v)mod3|,\;U_{B}=\sum_{j=0}^{2}e^{2\pi isj/3}|j\rangle \langle (j\;+\;t)mod3|,\;U_{C}=\sum_{l=0}^{2}e^{2\pi iml/3}|l\rangle \langle (l\;+\;n)mod3|.$$ (55)

Then the state $|{𝒬}^{\prime }\rangle$ as shown in Eq (54) changed to

$$|{𝒬}^{\prime \prime }\rangle =(U_{B}⊗U_{C}⊗U_{A})|{𝒬}^{\prime }\rangle \;=\{\sum_{j=0}^{2}\alpha_{j}(1\;-\;\gamma {)}^{\min[1,(j\;+\;t)mod3]}e^{i\theta_{j}}|j\rangle_{B_{1}}\}\;\;⊗\{\sum_{l=0}^{2}\beta_{l}(1\;-\;\gamma {)}^{\min[1,(l\;+\;n)mod3]}e^{i\vartheta_{l}}|l\rangle_{C_{1}}\}\;\;⊗\{\sum_{k=0}^{2}\lambda_{k}(1\;-\;\gamma {)}^{\min[1,(k\;+\;v)mod3]}e^{i\tau_{j}}|k\rangle_{A_{1}}\}.$$ (56)

($\tilde{\text{b}}$3) To proceed, Bob initializes an auxiliary qutrit *B*_2_ in the state $|0\rangle_{B_{2}}$. He then applies a unitary operation $U_{B}^{\prime }$ to the joint system composed of qutrit *B*_1_ and the auxiliary qutrit *B*_2_. When expressed in the computational basis $\{|00\rangle_{B_{1}B_{2}}$, $|01\rangle_{B_{1}B_{2}}$, $|10\rangle_{B_{1}B_{2}}$, $|11\rangle_{B_{1}B_{2}}$, $|20\rangle_{B_{1}B_{2}}$, $|21\rangle_{B_{1}B_{2}}\}$, the unitary operator $U_{B}^{\prime }$ corresponds to a 6×6 matrix. In the specific case where *t* = 0, the matrix form of $U_{B}^{\prime }$ is given by:

$$U_{B}^{\prime }=\left(\begin{array}{cccccc} 1\;-\;\gamma & \sqrt{1-(1\;-\;\gamma {)}^{2}} & 0 & 0 & 0 & 0\\ \sqrt{1-(1\;-\;\gamma {)}^{2}} & \gamma -1 & 0 & 0 & 0 & 0\\ 0 & 0 & 1 & 0 & 0 & 0\\ 0 & 0 & 0 & 1 & 0 & 0\\ 0 & 0 & 0 & 0 & 1 & 0\\ 0 & 0 & 0 & 0 & 0 & 1 \end{array}\right);$$ (57)

if *t* = 1,

$$U_{B}^{\prime }=\left(\begin{array}{cccccc} 1 & 0 & 0 & 0 & 0 & 0\\ 0 & 1 & 0 & 0 & 0 & 0\\ 0 & 0 & 1 & 0 & 0 & 0\\ 0 & 0 & 0 & 1 & 0 & 0\\ 0 & 0 & 0 & 0 & 1\;-\;\gamma & \sqrt{1-(1\;-\;\gamma {)}^{2}}\\ 0 & 0 & 0 & 0 & \sqrt{1-(1\;-\;\gamma {)}^{2}} & \gamma -1 \end{array}\right);$$ (58)

if *t* = 2,

$$U_{B}^{\prime }=\left(\begin{array}{cccccc} 1 & 0 & 0 & 0 & 0 & 0\\ 0 & 1 & 0 & 0 & 0 & 0\\ 0 & 0 & 1\;-\;\gamma & \sqrt{1-(1\;-\;\gamma {)}^{2}} & 0 & 0\\ 0 & 0 & \sqrt{1-(1\;-\;\gamma {)}^{2}} & \gamma -1 & 0 & 0\\ 0 & 0 & 0 & 0 & 1 & 0\\ 0 & 0 & 0 & 0 & 0 & 1 \end{array}\right).$$ (59)

After Bob’s unitary transformation $U_{B}^{\prime }$, the state $[\sum_{j=0}^{2}\alpha_{j}{\left(1\;-\;\gamma \right)}^{\min[1,(j\;+\;t)mod3]}e^{i\theta_{j}}|j\rangle {]}_{B_{1}}|0\rangle_{B_{2}}$ of qutrits *B*_1_ and *B*_2_ is transformed into

$$U_{B}^{\prime }\{\frac{1}{\sqrt{1\;+\;2(1\;-\;\gamma {)}^{2}}}[\sum_{j=0}^{2}\alpha_{j}(1\;-\;\gamma {)}^{\min[1,(j\;+\;t)mod3]}e^{i\theta_{j}}|j\rangle {]}_{B_{1}}|0\rangle_{B_{2}}\}\;=\frac{1\;-\;\gamma }{\sqrt{1\;+\;2(1\;-\;\gamma {)}^{2}}}[\alpha_{0}|0\rangle \;+\;\alpha_{1}e^{i\theta_{1}}|1\rangle \;+\;\alpha_{2}e^{i\theta_{2}}|2\rangle {]}_{B_{1}}|0\rangle_{B_{2}}\;\;\;+\;\frac{\sqrt{1-(1\;-\;\gamma {)}^{2}}}{\sqrt{1\;+\;2(1\;-\;\gamma {)}^{2}}}\alpha_{0}|0\rangle_{B_{1}}|1\rangle_{B_{2}}$$ (60)

or

$$U_{B}^{\prime }\{\frac{1}{\sqrt{1\;+\;2(1\;-\;\gamma {)}^{2}}}[\sum_{j=0}^{2}\alpha_{j}(1\;-\;\gamma {)}^{\min[1,(j\;+\;t)mod3]}e^{i\theta_{j}}|j\rangle {]}_{B_{1}}|0\rangle_{B_{2}}\}\;=\frac{1\;-\;\gamma }{\sqrt{1\;+\;2(1\;-\;\gamma {)}^{2}}}[\alpha_{0}|0\rangle \;+\;\alpha_{1}e^{i\theta_{1}}|1\rangle \;+\;\alpha_{2}e^{i\theta_{2}}|2\rangle {]}_{B_{1}}|0\rangle_{B_{2}}\;\;\;+\;\frac{\sqrt{1-(1\;-\;\gamma {)}^{2}}}{\sqrt{1\;+\;2(1\;-\;\gamma {)}^{2}}}\alpha_{2}e^{i\theta_{2}}|2\rangle_{B_{1}}|1\rangle_{B_{2}}$$ (61)

or

$$U_{B}^{\prime }\{\frac{1}{\sqrt{1\;+\;2(1\;-\;\gamma {)}^{2}}}[\sum_{j=0}^{2}\alpha_{j}(1\;-\;\gamma {)}^{\min[1,(j\;+\;t)mod3]}e^{i\theta_{j}}|j\rangle {]}_{B_{1}}|0\rangle_{B_{2}}\}\;=\frac{1\;-\;\gamma }{\sqrt{1\;+\;2(1\;-\;\gamma {)}^{2}}}[\alpha_{0}|0\rangle \;+\;\alpha_{1}e^{i\theta_{1}}|1\rangle \;+\;\alpha_{2}e^{i\theta_{2}}|2\rangle {]}_{B_{1}}|0\rangle_{B_{2}}\;\;\;+\;\frac{\sqrt{1-(1\;-\;\gamma {)}^{2}}}{\sqrt{1\;+\;2(1\;-\;\gamma {)}^{2}}}\alpha_{1}e^{i\theta_{1}}|1\rangle_{B_{1}}|1\rangle_{B_{2}}.$$ (62)

Subsequently, Bob performs a projective measurement on the auxiliary qutrit *B*_2_ in *Z*-basis. If the outcome is $|0\rangle_{B_{2}}$, it indicates that the CQT is successful, and Bob’s qutrit *B*_1_ collapses to Alice’s original unknown state $|\xi \rangle_{A}$. On the other hand, if the measurement result is $|1\rangle_{B_{2}}$, the cyclic QT is failed.

Based on Eqs (60), (61) and (62), we can determine the probability of Bob obtaining the outcome $|0\rangle_{B_{2}}$ as ${\left(1\;-\;\gamma \right)}^{2}/[\sum_{j=0}^{2}\alpha_{j}^{2}{\left(1\;-\;\gamma \right)}^{2\min[1,(j\;+\;t)mod3]}]$. Based on this, when Alice’s result is $|{ℬ}_{st}\rangle_{AA^{\prime }}$, the probability that Bob successfully reconstructs Alice’s original state can be expressed as ${\left(1\;-\;\gamma \right)}^{2}/[\sum_{j=0}^{2}\alpha_{j}^{2}{\left(1\;-\;\gamma \right)}^{2\min[1,(j\;+\;t)mod3]}]\;\times \;[\sum_{j=0}^{2}\alpha_{j}^{2}{\left(1\;-\;\gamma \right)}^{2\min[1,(j\;+\;t)mod3]}]/3[1\;+\;2{\left(1\;-\;\gamma \right)}^{2}]={\left(1\;-\;\gamma \right)}^{2}/3[1\;+\;2{\left(1\;-\;\gamma \right)}^{2}]$.

Similar to what Bob did in ($\tilde{\text{b}}$3), it is easy to see form Eq (54) that Charlie can reconstruct Bob’s original state $|\eta \rangle_{B}$ on his qubit *C*_1_ with probability ${\left(1\;-\;\gamma \right)}^{2}/2[1\;+\;2{\left(1\;-\;\gamma \right)}^{2}]$, and Alice can also reconstruct Charlie’s state $|\zeta \rangle_{C}$ on her qubit *A*_1_ with probability ${\left(1\;-\;\gamma \right)}^{2}/3[1\;+\;2{\left(1\;-\;\gamma \right)}^{2}]$. Therefore, the probability that Alice, Bob, and Charlie can successfully reconstruct the original states |ζ⟩, |ξ⟩ and |η⟩ when the measurement results $|{GB}_{st}\rangle_{AA^{\prime }}$, $|{GB}_{mn}\rangle_{BB^{\prime }}$ and $|{GB}_{uv}\rangle_{CC^{\prime }}$ occur simultaneously is ${\left(1\;-\;\gamma \right)}^{6}/27[1\;+\;2{\left(1\;-\;\gamma \right)}^{2}{]}^{3}$ i.e., for a fixed joint measurement outcome $|{GB}_{st}\rangle_{AA^{\prime }}|{GB}_{mn}\rangle_{BB^{\prime }}|{GB}_{uv}\rangle_{CC^{\prime }}$, the success probability of the cyclic QT is ${\left(1\;-\;\gamma \right)}^{6}/27[1\;+\;2{\left(1\;-\;\gamma \right)}^{2}{]}^{3}$. From the former analysis, s,t,m,n,u,v∈{0,1,2}, that is, Alice, Bob and Charlie each have 9 measurement outcomes. It means that the joint measurement outcomes they constitute have a total of 729, so the total probability of our CQT is $27{\left(1\;-\;\gamma \right)}^{6}/[1\;+\;2{\left(1\;-\;\gamma \right)}^{2}{]}^{3}$.

Note that when the noise strength of AD channel is zero (i.e., γ=0, there is no noise), then the states as shown in Eqs (49), (50) and (51) are all the same generalized Bell state $|{GB}_{00}\rangle$, and the probability of our scheme is $27{\left(1\;-\;\gamma \right)}^{6}/[1\;+\;2{\left(1\;-\;\gamma \right)}^{2}{]}^{3}=1$. It means that our scheme is the standard cyclic QT in three-dimensional quantum systems. In this sense, our CQT here is a generalization of the standard CQT.

**Stage 3** Clone unknown quantum states.

($\tilde{\text{c}}$1) In the third stage, Alice, Bob, and Charlie aim to independently create copies of arbitrary unknown single-qutrit states $|\xi \rangle_{A}$, $|\eta \rangle_{B}$ and $|\zeta \rangle_{C}$, respectively. To achieve this, they all require the assistance of the state preparer Victor.According to the projection assumption in quantum mechanics,if Alice, Bob and Charlie apply the generalized Bell-state measurement into |𝒬⟩ as shown in Eq (46), the state of qutrit pair $\left(A,A^{\prime }\right)$, $\left(B,B^{\prime }\right)$ and $\left(C,C^{\prime }\right)$ will collapse into some Bell states. For simplicity, we consider the collapsed state $|{GB}_{00}\rangle_{AA^{\prime }}$, $|{GB}_{00}\rangle_{B_{1}B^{\prime }}$ and $|{GB}_{00}\rangle_{CC^{\prime }}$. Following this collapse, each party sends one qutrit to Victor while retaining the other: Alice sends qutrit *A* to Victor and keeps $A^{\prime }$; Bob delivers *B* and retains $B^{\prime }$; and Charlie transmits *C* while keeping $C^{\prime }$. Given this setup, a natural question arises: can Victor, without any prior knowledge of the original input states, perform operations in the third stage that enable Alice, Bob, and Charlie to simultaneously recover copies of their respective unknown single-qutrit states? In what follows, we provide a detailed argument that such a process is indeed theoretically feasible.

($\tilde{\text{c}}$2) Notice that Victor completely knows the quantum state $|\xi \rangle_{A}$, $|\eta \rangle_{B}$ and $|\zeta \rangle_{C}$, so he can introduce three auxiliary qutrits $V_{0},V_{1}$ and *V*_2_, which are initially in the states $|0\rangle_{V_{0}}$, $|0\rangle_{V_{1}}$ and $|0\rangle_{V_{2}}$, respectively. Then, the composite system consisting of qutrits $A,A^{\prime },B,B^{\prime },C,C^{\prime }$, $V_{0},V_{1}$ and *V*_2_ is

$$|Q\rangle =|{GB}_{00}\rangle_{AA^{\prime }}|{GB}_{00}\rangle_{BB^{\prime }}|{GB}_{00}\rangle_{CC^{\prime }}|0\rangle_{V_{0}}|0\rangle_{V_{1}}|0\rangle_{V_{2}}.$$ (63)

Then he executes three unitary transformations $U_{0},U_{1}$ and *U*_2_, which are based on the bases $\{|00\rangle_{AV_{0}}$, $|01\rangle_{AV_{0}}$, $|10\rangle_{AV_{0}}$, $|11\rangle_{AV_{0}}$, $|20\rangle_{AV_{0}}$, $|21\rangle_{AV_{0}}\}$, $\{|00\rangle_{BV_{1}}$, $|01\rangle_{BV_{1}}$, $|10\rangle_{BV_{1}}$, $|11\rangle_{BV_{1}}$, $|20\rangle_{BV_{1}}$, $|21\rangle_{BV_{1}}\}$ and $\{|00\rangle_{CV_{2}}$, $|01\rangle_{CV_{2}}$, $|10\rangle_{CV_{2}}$, $|11\rangle_{CV_{2}}$, $|20\rangle_{CV_{2}}$, $|21\rangle_{CV_{2}}\}$, respectively, and have the following forms

$$U_{0}=\left(\begin{array}{cccccc} \alpha_{0} & \sqrt{1-\alpha_{0}^{2}} & 0 & 0 & 0 & 0\\ \sqrt{1-\alpha_{0}^{2}} & -\alpha_{0} & 0 & 0 & 0 & 0\\ 0 & 0 & \alpha_{1} & \sqrt{1-\alpha_{1}^{2}} & 0 & 0\\ 0 & 0 & \sqrt{1-\alpha_{1}^{2}} & -\alpha_{1} & 0 & 0\\ 0 & 0 & 0 & 0 & \alpha_{2} & \sqrt{1-\alpha_{2}^{2}}\\ 0 & 0 & 0 & 0 & \sqrt{1-\alpha_{1}^{2}} & -\alpha_{2} \end{array}\right),$$ (64)

$$U_{1}=\left(\begin{array}{cccccc} \beta_{0} & \sqrt{1-\beta_{0}^{2}} & 0 & 0 & 0 & 0\\ \sqrt{1-\beta_{0}^{2}} & -\beta_{0} & 0 & 0 & 0 & 0\\ 0 & 0 & \beta_{1} & \sqrt{1-\beta_{1}^{2}} & 0 & 0\\ 0 & 0 & \sqrt{1-\beta_{1}^{2}} & -\beta_{1} & 0 & 0\\ 0 & 0 & 0 & 0 & \beta_{2} & \sqrt{1-\beta_{2}^{2}}\\ 0 & 0 & 0 & 0 & \sqrt{1-\beta_{1}^{2}} & -\beta_{2} \end{array}\right),$$ (65)

$$U_{3}=\left(\begin{array}{cccccc} \lambda_{0} & \sqrt{1-\lambda_{0}^{2}} & 0 & 0 & 0 & 0\\ \sqrt{1-\lambda_{0}^{2}} & -\lambda_{0} & 0 & 0 & 0 & 0\\ 0 & 0 & \lambda_{1} & \sqrt{1-\lambda_{1}^{2}} & 0 & 0\\ 0 & 0 & \sqrt{1-\lambda_{1}^{2}} & -\lambda_{1} & 0 & 0\\ 0 & 0 & 0 & 0 & \lambda_{2} & \sqrt{1-\lambda_{2}^{2}}\\ 0 & 0 & 0 & 0 & \sqrt{1-\lambda_{1}^{2}} & -\lambda_{2} \end{array}\right).$$ (66)

After these unitary transformations, the state |Q⟩ becomes

$$|Q^{\prime }\rangle =(U_{0}⊗U_{1}⊗U_{2})|Q\rangle \;=\frac{1}{3\sqrt{3}}[(\alpha_{0}|00\rangle \;+\;\alpha_{1}|11\rangle \;+\;\alpha_{2}|22\rangle {)}_{AA^{\prime }}|0\rangle_{V_{0}}\;\;\;+\;(\sqrt{1-\alpha_{0}^{2}}|00\rangle \;+\;\sqrt{1-\alpha_{1}^{2}}|11\rangle \;+\;\sqrt{1-\alpha_{2}^{2}}|22\rangle {)}_{AA^{\prime }}|1\rangle_{V_{0}}]\;\;⊗[(\beta_{0}|00\rangle \;+\;\beta_{1}|11\rangle \;+\;\beta_{2}|22\rangle {)}_{BB^{\prime }}|0\rangle_{V_{1}}\;\;\;+\;(\sqrt{1-\beta_{0}^{2}}|00\rangle \;+\;\sqrt{1-\beta_{1}^{2}}|11\rangle \;+\;\sqrt{1-\beta_{2}^{2}}|22\rangle {)}_{BB^{\prime }}|1\rangle_{V_{1}}]\;\;⊗[(\lambda_{0}|00\rangle \;+\;\lambda_{1}|11\rangle \;+\;\lambda_{2}|22\rangle {)}_{CC^{\prime }}|0\rangle_{V_{2}}\;\;\;+\;(\sqrt{1-\lambda_{0}^{2}}|00\rangle \;+\;\sqrt{1-\lambda_{1}^{2}}|11\rangle \;+\;\sqrt{1-\lambda_{2}^{2}}|22\rangle {)}_{CC^{\prime }}|1\rangle_{V_{2}}].\;$$ (67)

Now, Victor performs projective measurements on the auxiliary qutrits $V_{0},V_{1}$ and *V*_2_, respectively, under the basis {|0⟩,|1⟩}. If the joint result is $|0\rangle_{V_{0}}|0\rangle_{V_{1}}|0\rangle_{V_{2}}$ with the probability of 1/9, the state of the system composed of qutrits $A,A^{\prime },B,B^{\prime },C$ and $C^{\prime }$ collapses into

$$|Q^{\prime \prime }\rangle =(\alpha_{0}|00\rangle \;+\;\alpha_{1}|11\rangle \;+\;\alpha_{2}|22\rangle {)}_{AA^{\prime }}\;\;⊗(\beta_{0}|00\rangle \;+\;\beta_{1}|11\rangle \;+\;\beta_{2}|22\rangle {)}_{BB^{\prime }}\;\;⊗(\lambda_{0}|00\rangle \;+\;\lambda_{1}|11\rangle \;+\;\lambda_{2}|22\rangle {)}_{CC^{\prime }}.$$ (68)

Otherwise it fail.

($\tilde{\text{c}}$3) Let us introduce the following notations:

$${𝒲}_{1}=\frac{1}{\sqrt{3}}\left(\begin{array}{ccc} 1 & e^{-i\theta_{1}} & e^{-i\theta_{2}}\\ 1 & e^{i(2\pi /3-\theta_{1})} & e^{i(4\pi /3-\theta_{2})}\\ 1 & e^{i(4\pi /3-\theta_{1})} & e^{i(2\pi /3-\theta_{2})} \end{array}\right),\;{𝒲}_{2}=\frac{1}{\sqrt{3}}\left(\begin{array}{ccc} 1 & e^{-i\vartheta_{1}} & e^{-i\vartheta_{2}}\\ 1 & e^{i(2\pi /3-\vartheta_{1})} & e^{i(4\pi /3-\vartheta_{2})}\\ 1 & e^{i(4\pi /3-\vartheta_{1})} & e^{i(2\pi /3-\vartheta_{2})} \end{array}\right),\;{𝒲}_{3}=\frac{1}{\sqrt{3}}\left(\begin{array}{ccc} 1 & e^{-i\tau_{1}} & e^{-i\tau_{2}}\\ 1 & e^{i(2\pi /3-\tau_{1})} & e^{i(4\pi /3-\tau_{2})}\\ 1 & e^{i(4\pi /3-\tau_{1})} & e^{i(2\pi /3-\tau_{2})} \end{array}\right).$$ (69)

Obviously, the vectors in each group in Eq (69) form a complete orthogonal basis within a 3-dimensional Hilbert space.

Given that Victor has full knowledge of the unknown states $|\xi \rangle_{A}$, $|\eta \rangle_{B}$ and $|\zeta \rangle_{C}$,he performs a measurement on qutrits *A*, *B*, and *C* using a specially constructed basis denoted by $\left\{|\varepsilon_{rst}\rangle_{ABC}|r,s,t=0,1,2\right\}$.This measurement basis is related to the standard three-qutrit computational basis through the following transformation:

$$\left(\begin{array}{c} |\varepsilon_{000}\rangle \\ |\varepsilon_{001}\rangle \\ \vdots \\ |\varepsilon_{222}\rangle \end{array}\right)=({𝒲}_{1}⊗{𝒲}_{2}⊗{𝒲}_{3})\left(\begin{array}{c} |000\rangle \\ |001\rangle \\ \vdots \\ |222\rangle \end{array}\right),$$ (70)

Generally, if Victor’s measurement outcome is $|\varepsilon_{rst}\rangle_{ABC}$, the collapsed state of qutrits $A^{\prime }$, $B^{\prime }$ and $C^{\prime }$ is

$${\;}_{ABC}\langle \varepsilon_{rst}|Q^{\prime \prime }\rangle =\frac{1}{3\sqrt{3}}(\alpha_{0}|0\rangle \;+\;e^{-2\pi r/3}\alpha_{1}e^{i\theta_{1}}|1\rangle \;+\;e^{-4\pi r/3}\alpha_{2}e^{i\theta_{2}}|2\rangle {)}_{A^{\prime }}\;\;⊗(\beta_{0}|0\rangle \;+\;e^{-2\pi s/3}\alpha_{1}e^{i\vartheta_{1}}|1\rangle \;+\;e^{-4\pi s/3}\beta_{2}e^{i\vartheta_{2}}|2\rangle {)}_{B^{\prime }}\;\;⊗(\lambda_{0}|0\rangle \;+\;e^{-2\pi t/3}\lambda_{1}e^{i\tau_{1}}|1\rangle \;+\;e^{-4\pi t/3}\lambda_{2}e^{i\tau_{2}}|2\rangle {)}_{C^{\prime }}.$$ (71)

After conducting a projective measurement on qutrits *A*,*B* and *C*, Victor subsequently communicates the measurement outcome to Alice, Bob, and Charlie through classical communication channels.

($\tilde{\text{c}}$4) Upon receiving the measurement information from Victor, each of Alice, Bob, and Charlie is able to apply a corresponding local unitary transformation to recover their respective target states. Specifically, for the above general measurement result $|\varepsilon_{rst}\rangle_{ABC}$, Alice, Bob and Charlie perform unitary transformations ${\bar{U}}_{A},{\bar{U}}_{B}$ and ${\bar{U}}_{C}$ on their respective qutrits, which given as follows

$${\bar{U}}_{A}=|0\langle 0|\;+\;e^{2\pi r/3}|1\rangle \langle 1|\;+\;e^{4\pi r/3}|2\rangle \langle 2|,\;{\bar{U}}_{B}=|0\langle 0|\;+\;e^{2\pi s/3}|1\rangle \langle 1|\;+\;e^{4\pi s/3}|2\rangle \langle 2|,\;{\bar{U}}_{C}=|0\langle 0|\;+\;e^{2\pi t/3}|1\rangle \langle 1|\;+\;e^{4\pi t/3}|2\rangle \langle 2|,$$ (72)

i.e.,

$$({\bar{U}}_{A}⊗{\bar{U}}_{B}⊗{\bar{U}}_{C})({\;}_{ABC}\langle \varepsilon_{rst}|Q^{\prime \prime }\rangle )=\frac{1}{3\sqrt{3}}(\alpha_{0}|0\rangle \;+\;\alpha_{1}e^{i\theta_{1}}|1\rangle \;+\;\alpha_{2}e^{i\theta_{2}}|2\rangle {)}_{A^{\prime }}\;\;⊗(\beta_{0}|0\rangle \;+\;\alpha_{1}e^{i\vartheta_{1}}|1\rangle \;+\;\beta_{2}e^{i\vartheta_{2}}|2\rangle {)}_{B^{\prime }}\;\;⊗(\lambda_{0}|0\rangle \;+\;\lambda_{1}e^{i\tau_{1}}|1\rangle \;+\;\lambda_{2}e^{i\tau_{2}}|2\rangle {)}_{C^{\prime }},\;=\frac{1}{3\sqrt{3}}|\xi \rangle_{A^{\prime }}⊗|\eta \rangle_{B^{\prime }}⊗|\zeta \rangle_{C^{\prime }},$$ (73)

completing the cloning task.

By combining the method presented in Sect 3.2 with the three-party cyclic-assisted cloning scheme proposed in this section, we can further extend the protocol to accommodate *N*-party cyclic-assisted cloning, where *N*>3.

## 5 Cyclic assisted cloning of arbitrary unknown single-qudit states in amplitude damping channel

In this section, we extend the schemes from the previous two sections to the problem of cyclic-assisted cloning of high-dimensional unknown single-particle states in the AD channel. Firstly, we still consider the three-party assisted cloning: suppose that there are four participants, Alice, Bob, Charlie, and Victor, spatially separated. The state preparer Victor has prepared three single-qudit states $|\phi \rangle_{A}$, $|\varphi \rangle_{B}$ and $|\psi \rangle_{C}$, as follows

$$|\phi \rangle_{A}=\sum_{j=0}^{d\;-\;1}\alpha_{j}e^{i\theta_{j}}|j\rangle_{A},\;|\varphi \rangle_{B}=\sum_{j=0}^{d\;-\;1}\beta_{j}e^{i\vartheta_{j}}|j\rangle_{B},\;|\psi \rangle_{C}=\sum_{j=0}^{d\;-\;1}\lambda_{j}e^{i\tau_{j}}|j\rangle_{C},$$ (74)

where $\alpha_{j}$, $\beta_{j}$ and $\lambda_{j}$ (j=0,1,⋯,d−1) are real numbers with $\sum_{j=0}^{d\;-\;1}\alpha_{j}^{2}=1$, $\sum_{j=0}^{d\;-\;1}\beta_{j}^{2}=1$ and $\sum_{j=0}^{d\;-\;1}\lambda_{j}^{2}=1$, while $\theta_{j}$, $\vartheta_{j}$, $\tau_{j}$ are arbitrary real numbers for any j∈{1,2,⋯,d−1} and $\theta_{0}=\vartheta_{0}=\tau_{0}=0$. All the parameters of the states in Eq (74) are completely known to Victor and completely unknown to the other participants. Victor distributes the quantum states *A*, *B*, and *C* to Alice, Bob, and Charlie, respectively. Upon receiving these states, each of them treats the received state as their own input. Importantly, none of the participants,except for Victor,has any knowledge of these input states. The goal of Alice, Bob, and Charlie is to sequentially teleport their individual input states to Bob, Charlie, and Alice, respectively. With assistance from Victor, they aim to reproduce the original input states at their own locations, thereby generating copies of these states.The schematic diagram of the scheme presented in this section is highly similar to that of the scheme in Sect 3. The protocol in Sect 3 involves the cyclic assisted cloning of arbitrary unknown single-qubit states in an amplitude damping channel, while this section explores the cyclic assisted cloning of arbitrary unknown single-qudit states in the same channel. This represents an extension of the protocol in Sect 3 to the scenario of cyclic assisted cloning for d-dimensional unknown quantum states. The main difference lies in the type of quantum states being transmitted (single-qubit states vs. single-qudit states), while the cyclic transmission method and Victor’s assistance mechanism remain unchanged. To avoid redundancy, no additional diagrams are included here to enhance the clarity of the content. This scheme mainly includes three stages: preparation of quantum channels, CQT and assisted cloning.

In the preparation stage of the quantum channels, in a similar method to that in Sects 3.1 and 4, Alice and Bob share a *d*-dimensional two-particle PES

$$|H\rangle_{A^{\prime }B_{1}}=\frac{1}{\sqrt{1\;+\;(d\;-\;1)(1\;-\;\gamma {)}^{2}}}[|00\rangle \;+\;(1\;-\;\gamma )|11\rangle \;+\;\cdots \;+\;(1\;-\;\gamma )|d\;-\;1,d\;-\;1\rangle {]}_{A^{\prime }B_{1}},$$ (75)

Bob and Charlie share a two-qudit PES

$$|H\rangle_{B^{\prime }C_{1}}=\frac{1}{\sqrt{1\;+\;(d\;-\;1)(1\;-\;\gamma {)}^{2}}}[|00\rangle \;+\;(1\;-\;\gamma )|11\rangle \;+\;\cdots \;+\;(1\;-\;\gamma )|d\;-\;1,d\;-\;1\rangle {]}_{B^{\prime }C_{1}},$$ (76)

and Charlie and Alice share a two-qudit PES

$$|H\rangle_{C^{\prime }A_{1}}=\frac{1}{\sqrt{1\;+\;(d\;-\;1)(1\;-\;\gamma {)}^{2}}}[|00\rangle \;+\;(1\;-\;\gamma )|11\rangle \;+\;\cdots \;+\;(1\;-\;\gamma )|d\;-\;1,d\;-\;1\rangle {]}_{C^{\prime }A_{1}}.$$ (77)

Therefore, the state of the composite system of qudits $A,A^{\prime },A_{1},B,B^{\prime },B_{1},C,C^{\prime }$ and *C*_1_ is

$$|𝒯\rangle =|\phi \rangle_{A}⊗|\varphi \rangle_{B}⊗|\psi \rangle_{C}⊗|H\rangle_{A^{\prime }B_{1}}⊗|H\rangle_{B^{\prime }C_{1}}⊗|H\rangle_{C^{\prime }A_{1}}.$$ (78)

Now consider the cyclic QT: Alice, Bob and Charlie perform *d*-dimensional Bell measurements on qudit pairs $\left(A,A^{\prime }\right)$, $\left(B,B^{\prime }\right)$,and $\left(C,C^{\prime }\right)$, and communicate the results $|{DB}_{st}\rangle_{AA^{\prime }}$, $|{DB}_{mn}\rangle_{BB^{\prime }}$ and $|{DB}_{uv}\rangle_{CC^{\prime }}$ to Bob, Charlie and Alice in order via the classical channels. After these measurements, the state of $B_{1},C_{1}$ and *A*_1_ will collapse into

$$\;{\;}_{CC^{\prime }}\langle {DB}_{uv}|{\;}_{BB^{\prime }}\langle {DB}_{mn}|{\;}_{AA^{\prime }}\langle {DB}_{st}|𝒯\rangle \;=\frac{1}{\sqrt{[d\;+\;d(d\;-\;1)(1\;-\;\gamma {)}^{2}{]}^{3}}}\sum_{j=0}^{d\;-\;1}\alpha_{j}e^{i\theta_{j}-2\pi ijs/d}(1\;-\;\gamma {)}^{\min[1,(j\;+\;t)modd]}|(j\;+\;t)modd\rangle_{B_{1}}\;\;⊗\sum_{l=0}^{d\;-\;1}\beta_{l}e^{i\vartheta_{l}-2\pi ilm/d}(1\;-\;\gamma {)}^{\min[1,(l\;+\;n)modd]}|(l\;+\;n)modd\rangle_{C_{1}}\;\;⊗\sum_{k=0}^{d\;-\;1}\lambda_{k}e^{i\tau_{k}-2\pi iku/d}(1\;-\;\gamma {)}^{\min[1,(k\;+\;v)modd]}|(k\;+\;v)modd\rangle_{A_{1}}.$$ (79)

According to the measurement information, Bob, Charlie and Alice apply the local unitary operations ${𝒰}_{B}$, ${𝒰}_{C}$ and ${𝒰}_{A}$ on qudits $B_{1},C_{1}$ and *A*_1_, respectively, which are given by

$${𝒰}_{B}=\sum_{j=0}^{d\;-\;1}e^{2\pi ijs/d}|j\rangle \langle (j\;+\;t)modd\rangle ,\;{𝒰}_{C}=\sum_{l=0}^{d\;-\;1}e^{2\pi ilm/d}|l\rangle \langle (l\;+\;n)modd\rangle ,\;{𝒰}_{A}=\sum_{k=0}^{d\;-\;1}e^{2\pi iku/d}|k\rangle \langle (k\;+\;v)modd\rangle .\;$$ (80)

These local unitary operations change the state ${\;}_{CC^{\prime }}\langle {DB}_{uv}|{\;}_{BB^{\prime }}\langle {DB}_{mn}|{\;}_{AA^{\prime }}\langle {DB}_{st}|𝒯\rangle$ into

$$|{𝒯}^{\prime }\rangle =\frac{1}{\sqrt{[d\;+\;d(d\;-\;1)(1\;-\;\gamma {)}^{2}{]}^{3}}}\sum_{j=0}^{d\;-\;1}\alpha_{j}e^{i\theta_{j}}(1\;-\;\gamma {)}^{\min[1,(j\;+\;t)modd]}|j\rangle_{B_{1}}\;\;⊗\sum_{l=0}^{d\;-\;1}\beta_{l}e^{i\vartheta_{l}}(1\;-\;\gamma {)}^{\min[1,(l\;+\;n)modd]}|l\rangle_{C_{1}}\;\;⊗\sum_{k=0}^{d\;-\;1}\lambda_{k}e^{i\tau_{k}}(1\;-\;\gamma {)}^{\min[1,(k\;+\;v)modd]}|k\rangle_{A_{1}}.$$ (81)

To proceed, Alice prepares an auxiliary qubit *A*_2_ initialized in the state $|0\rangle_{A_{2}}$. She then applies a unitary transformation, denoted as ${𝒰}_{A}^{\prime }$, which operates in the basis $\left\{|j0\rangle_{A_{1}A_{2}},|j1\rangle_{A_{1}A_{2}}\;|\;j=0,1,\ldots ,d\;-\;1\right\}$, which is given by

$${𝒰}_{A}^{\prime }=diag(\underset{d-v}{\underbrace{E,\cdots ,E}},W_{v},\underset{v-1}{\underbrace{E,\cdots ,E}}),v=1,2,\cdots ,d\;-\;1$$ (82)

and when v=0,

$${𝒰}_{A}^{\prime }=diag(W_{0},\underset{d\;-\;1}{\underbrace{E,\cdots ,E}}),$$ (83)

where *E* represents the 2×2 identity matrix, and

$$W_{v}\equiv \left(\begin{array}{cc} 1\;-\;\gamma & \sqrt{1-(1\;-\;\gamma {)}^{2}}\\ \sqrt{1-(1\;-\;\gamma {)}^{2}} & \gamma -1 \end{array}\right)$$ (84)

for any v∈{0,1,⋯,d−1}. Then,it is straightforward to deduce that

$$\;{𝒰}_{A}^{\prime }[\frac{1}{\sqrt{d\;+\;d(d\;-\;1)(1\;-\;\gamma {)}^{2}}}\sum_{k=0}^{d\;-\;1}\lambda_{k}e^{i\tau_{k}}(1\;-\;\gamma {)}^{\min[1,(k\;+\;v)modd]}|k\rangle_{A_{1}}|0\rangle_{A_{2}}]\;=\frac{1\;-\;\gamma }{\sqrt{d\;+\;d(d\;-\;1)(1\;-\;\gamma {)}^{2}}}[\sum_{k=0}^{d\;-\;1}\lambda_{k}e^{i\tau_{k}}|k\rangle_{A_{1}}]|0\rangle_{A_{2}}\;\;\;+\;\frac{\sqrt{1-(1\;-\;\gamma {)}^{2}}}{\sqrt{d\;+\;d(d\;-\;1)(1\;-\;\gamma {)}^{2}}}\lambda_{(d-v)modd}e^{i\tau_{(d-v)modd}}|(d-v)modd\rangle_{A_{1}}|1\rangle_{A_{2}}$$ (85)

for any v∈{0,1,⋯,d−1}. Now Alice performs a projective measurement on the auxiliary qubit *A*_2_ in the basis {|0⟩,|1⟩}. If theresult of Alice’s measurement is $|0\rangle_{A_{2}}$, she successfully retrieves Charlie’s state $|\psi \rangle_{C}$ on her qudit *A*_1_. Otherwise, the teleportation fails.

Clearly, the probability of obtaining the measurement result $|0\rangle_{A_{2}}$ is given by


$$(1\;-\;\gamma {)}^{2}/[\sum_{k=0}^{d\;-\;1}\lambda_{k}^{2}(1\;-\;\gamma {)}^{2\min[1,(k\;+\;v)modd]}].$$

Form the above analysis, on condition that Charlie’s result is $|{DB}_{uv}\rangle_{CC^{\prime }}$ with the probability of $\sum_{k=0}^{d\;-\;1}\lambda_{k}^{2}{\left(1\;-\;\gamma \right)}^{2\min[1,(k\;+\;v)modd]}/d[1\;+\;(d\;-\;1){\left(1\;-\;\gamma \right)}^{2}]$, it can be observed that the probability of Alice successfully recovering Charlie’s original state is given by ${\left(1\;-\;\gamma \right)}^{2}/[\sum_{k=0}^{d\;-\;1}\lambda_{k}^{2}{\left(1\;-\;\gamma \right)}^{2\min[1,(k\;+\;v)modd]}]\;\times \;\sum_{k=0}^{d\;-\;1}\lambda_{k}^{2}{\left(1\;-\;\gamma \right)}^{2\min[1,(k\;+\;v)modd]}/d[1\;+\;(d\;-\;1)$ ${\left(1\;-\;\gamma \right)}^{2}]={\left(1\;-\;\gamma \right)}^{2}/d[1\;+\;(d\;-\;1){\left(1\;-\;\gamma \right)}^{2}]$.

Similar to Alice’s approach, Bob and Charlie independently reconstruct the original states of Alice and Bob, respectively, with the probability of ${\left(1\;-\;\gamma \right)}^{2}/d[1\;+\;(d\;-\;1){\left(1\;-\;\gamma \right)}^{2}]$. Since s,t,m,n,u,v=0,1,⋯,d−1, the overall success probability of the cyclic quantum teleportation is $d^{3}{\left(1\;-\;\gamma \right)}^{6}/[1\;+\;(d\;-\;1){\left(1\;-\;\gamma \right)}^{2}{]}^{3}$.

In the assisted cloning stage, based on the projection postulate of quantum mechanics, if Alice, Bob and Charlie apply the *d*-dimensional Bell state measurements onto the combined state |𝒯⟩, the state of qudit pairs pair $\left(A,A^{\prime }\right)$, pair $\left(B,B^{\prime }\right)$ and pair $\left(C,C^{\prime }\right)$ will collapse into some *d*-dimensional Bell states. Without loss of generality, we consider the following three collapsed Bell states $|{DB}_{00}\rangle_{AA^{\prime }}$, $|{DB}_{00}\rangle_{BB^{\prime }}$ and $|{DB}_{00}\rangle_{CC^{\prime }}$. Alice transmits qudit *A* to Victor while retaining qudit $A^{\prime }$ herself; similarly, Bob sends qudit *B* to Victor and keeps qudit $B^{\prime }$ in his possession; likewise, Charlie forwards qudit *C* to Victor and holds onto qudit $C^{\prime }$.

Since Victor knows exactly the three quantum states indicated in Eq (74), he introduces three auxiliary qubits $V^{\prime },V^{\prime \prime }$ and $V^{\prime },V^{\prime \prime }$ in initial states $|0\rangle_{V^{\prime }},|0\rangle_{V^{\prime \prime }}$ and $|0\rangle_{V^{\prime }},|0\rangle_{V^{\prime \prime }}$, respectively. Then, the composite system consisting of qudits $A,A^{\prime },B,B^{\prime },C,C^{\prime }$, $V^{\prime },V^{\prime \prime }$ and $V^{\prime },V^{\prime \prime }$ is

$$|T\rangle =|{\mathrm{DB}}_{00}\rangle_{AA'}|{\mathrm{DB}}_{00}\rangle_{BB'}|{\mathrm{DB}}_{00}\rangle_{CC'}|0\rangle_{V'}|0\rangle_{V''}|0\rangle_{V'''},$$ (86)

and then, he performs three unitary transformations ${\text{U}}^{\left(0\right)},{\text{U}}^{\left(1\right)}$ and ${\text{U}}^{\left(2\right)}$, which are based on the bases $\{|j0\rangle_{AV^{\prime }}$, $|j1\rangle_{AV^{\prime }}:j=0,1,\cdots ,d\;-\;1\}$, $\{|l0\rangle_{BV^{\prime \prime }}$, $|l1\rangle_{BV^{\prime \prime }}:l=0,1,\cdots ,d\;-\;1\}$ and $|l1\rangle_{BV^{\prime \prime }}:l=0,1,\cdots ,d\;-\;1\}$, $|l1\rangle_{BV^{\prime \prime }}:l=0,1,\cdots ,d\;-\;1\}$, respectively,and are expressed as follows

$$U^{\left(0\right)}=diag(U_{0,0}^{\left(0\right)},U_{0,1}^{\left(0\right)},\cdots ,U_{0,j}^{\left(0\right)},\cdots ,U_{0,d\;-\;1}^{\left(0\right)}),\;U^{\left(1\right)}=diag(U_{0,0}^{\left(1\right)},U_{0,1}^{\left(1\right)},\cdots ,U_{0,l}^{\left(1\right)},\cdots ,U_{0,d\;-\;1}^{\left(1\right)}),\;U^{\left(2\right)}=diag(U_{0,0}^{\left(2\right)},U_{0,1}^{\left(2\right)},\cdots ,U_{0,k}^{\left(2\right)},\cdots ,U_{0,d\;-\;1}^{\left(2\right)}),\;$$ (87)

where

$$U_{0,j}^{\left(0\right)}=\left(\begin{array}{cc} \alpha_{j} & \sqrt{1-\alpha_{j}^{2}}\\ \sqrt{1-\alpha_{j}^{2}} & -\alpha_{j} \end{array}\right),\;U_{0,l}^{\left(1\right)}=\left(\begin{array}{cc} \beta_{l} & \sqrt{1-\beta_{l}^{2}}\\ \sqrt{1-\beta_{l}^{2}} & -\beta_{l} \end{array}\right),\;U_{0,k}^{\left(2\right)}=\left(\begin{array}{cc} \lambda_{k} & \sqrt{1-\lambda_{k}^{2}}\\ \sqrt{1-\lambda_{k}^{2}} & -\alpha_{k} \end{array}\right).$$ (88)

After these unitary transformations, the state |T⟩ becomes

$$|T'\rangle =(U^{\left(0\right)}⊗U^{\left(1\right)}⊗U^{\left(2\right)})|T\rangle \;=\frac{1}{d\sqrt{d}}[\sum_{j=0}^{d\;-\;1}\alpha_{j}|jj\rangle_{AA'}|0\rangle_{V'}\;+\;\sum_{j=0}^{d\;-\;1}\sqrt{1-\alpha_{j}^{2}}|jj\rangle_{AA'}|1\rangle_{V'}]\;\;\;⊗[\sum_{l=0}^{d\;-\;1}\beta_{l}|ll\rangle_{BB'}|0\rangle_{V''}\;+\;\sum_{l=0}^{d\;-\;1}\sqrt{1-\beta_{l}^{2}}|ll\rangle_{BB'}|1\rangle_{V''}]\;\;\;⊗[\sum_{k=0}^{d\;-\;1}\lambda_{k}|kk\rangle_{CC'}|0\rangle_{V'''}\;+\;\sum_{k=0}^{d\;-\;1}\sqrt{1-\lambda_{k}^{2}}|kk\rangle_{CC'}|1\rangle_{V'''}].$$ (89)

Subsequently, Victor respectively measures the auxiliary qubits $V^{\prime },V^{\prime \prime }$ and $V^{\prime },V^{\prime \prime }$ in the computational basis {|0⟩,|1⟩}. If the collective measurement outcome is {|0⟩,|1⟩}, then the state of qudits $A,A^{\prime },B,B^{\prime },C$ and $C^{\prime }$ collapses accordingly into

$$|T^{\prime \prime }\rangle =\frac{1}{d\sqrt{d}}[\sum_{j=0}^{d\;-\;1}\alpha_{j}|jj\rangle_{AA^{\prime }}]⊗[\sum_{l=0}^{d\;-\;1}\beta_{l}|ll\rangle_{BB^{\prime }}]⊗[\sum_{k=0}^{d\;-\;1}\lambda_{k}|kk\rangle_{CC^{\prime }}].$$ (90)

Let us introduces the Matrix notation

$$\;𝒲(\theta_{1},\theta_{2},\cdots ,\theta_{d\;-\;1})\;=\frac{1}{\sqrt{d}}\left(\begin{array}{ccccc} 1 & e^{-i\theta_{1}} & e^{-i\theta_{2}} & \cdots & e^{-i\theta_{d\;-\;1}}\\ 1 & e^{i(2\pi /d-\theta_{1})} & e^{i(4\pi /d-\theta_{2})} & \cdots & e^{i[2(d\;-\;1)\pi /d-\theta_{d\;-\;1}]}\\ 1 & e^{i(4\pi /d-\theta_{1})} & e^{i(8\pi /d-\theta_{2})} & \cdots & e^{i[4(d\;-\;1)\pi /d-\theta_{d\;-\;1}]}\\ \cdots & \cdots & \cdots & \cdots & \cdots \\ 1 & e^{i[2(d\;-\;1)\pi /d-\theta_{1}]} & e^{i[4(d\;-\;1)\pi /d-\theta_{2}]} & \cdots & e^{i[2(d\;-\;1{)}^{2}\pi /d-\theta_{d\;-\;1}]} \end{array}\right).$$ (91)

Since Victor has complete knowledge of the states $|\phi \rangle_{A}$, $|\varphi \rangle_{B}$, and $|\psi \rangle_{C}$, he performs a measurement on qudits *A*, *B*, and *C* using the basis $\left\{|\varepsilon_{rst}\rangle_{ABC}:r,s,t=0,1,\cdots ,d\;-\;1\right\}$.The correspondence between this measurement basis and the standard computational basis of the three-qudit system is defined as follows

$$\left(\begin{array}{c} |\varepsilon_{000}\rangle \\ |\varepsilon_{001}\rangle \\ \vdots \\ |\varepsilon_{d\;-\;1,d\;-\;1,d\;-\;1}\rangle \end{array}\right)=𝒲(\theta_{1},\theta_{2},\cdots ,\theta_{d\;-\;1})⊗𝒲(\vartheta_{1},\vartheta_{2},\cdots ,\vartheta_{d\;-\;1})\;⊗𝒲(\tau_{1},\tau_{2},\cdots ,\tau_{d\;-\;1})\left(\begin{array}{c} |000\rangle \\ |001\rangle \\ \vdots \\ |d\;-\;1,d\;-\;1,d\;-\;1\rangle \end{array}\right).$$ (92)

In general, if Victor’s measurement results in the outcome $|\varepsilon_{rst}\rangle_{ABC}$, the corresponding post-measurement state of qutrits $A^{\prime }$, $B^{\prime }$, and $C^{\prime }$ is given by the following expression

$${\;}_{ABC}\langle \varepsilon_{rst}|T^{\prime \prime }\rangle =\frac{1}{d\sqrt{d}}[\sum_{j=0}^{d\;-\;1}\alpha_{j}e^{i(\theta_{j}-2\pi jr/d)}|j\rangle_{A^{\prime }}]\;\;⊗[\sum_{l=0}^{d\;-\;1}\beta_{l}e^{i(\vartheta_{l}-2\pi ls/d)}|l\rangle_{B^{\prime }}]\;\;⊗[\sum_{k=0}^{d\;-\;1}\lambda_{k}e^{i(\tau_{k}-2\pi kt/d)}|k\rangle_{C^{\prime }}].$$ (93)

Following a projective measurement performed by Victor on the qutrits *A*, *B*, and *C*, he communicates the measurement result to Alice, Bob, and Charlie through classical communication channels. Based on the information received from Victor, each of them can apply a suitable local unitary operation to retrieve their respective intended quantum states. Specifically,for the above general measurement result $|\varepsilon_{rst}\rangle_{ABC}$, Alice, Bob, and Charlie apply the unitary operators ${\dot{U}}_{A}$, ${\dot{U}}_{B}$, and ${\dot{U}}_{C}$ to their corresponding qudits. These operations are defined as follows:

$${\dot{U}}_{A}=\sum_{j=0}^{d\;-\;1}e^{2\pi ijr/d}|j\rangle \langle j{|}_{A^{\prime }},\;{\dot{U}}_{B}=\sum_{l=0}^{d\;-\;1}e^{2\pi ils/d}|l\rangle \langle l{|}_{B^{\prime }},\;{\dot{U}}_{C}=\sum_{k=0}^{d\;-\;1}e^{2\pi ikt/d}|k\rangle \langle k{|}_{C^{\prime }},$$ (94)

i.e.,

$$\;({\dot{U}}_{A}⊗{\dot{U}}_{B}⊗{\dot{U}}_{C})({\;}_{ABC}\langle \varepsilon_{rst}|T^{\prime \prime }\rangle )\;=\frac{1}{d\sqrt{d}}(\sum_{j=0}^{d\;-\;1}\alpha_{j}e^{i\theta_{j}}|j\rangle_{A^{\prime }})⊗(\sum_{l=0}^{d\;-\;1}\beta_{l}e^{i\vartheta_{l}}|l\rangle_{B^{\prime }})⊗(\sum_{k=0}^{d\;-\;1}\lambda_{k}e^{i\tau_{k}}|k\rangle_{C^{\prime }})\;=\frac{1}{d\sqrt{d}}|\phi \rangle_{A^{\prime }}⊗|\varphi \rangle_{B^{\prime }}⊗|\psi \rangle_{C^{\prime }}.$$ (95)

This means that arbitrary unknown single-qudit states $|\phi \rangle_{A}$, $|\varphi \rangle_{B}$ and $|\psi \rangle_{C}$ are perfectly copied at the locations where Alice, Bob, and Charlie are, respectively, with the assistance of the state preparer Victor, completing the cloning task.

By integrating the concept introduced in Sect 3.2 with the cyclic-assisted cloning strategy involving three participants presented in this section, the proposed scheme can be naturally extended to accommodate a cyclic-assisted cloning protocol among *N* parties, where *N*>3.

## 6 Discussion and conclusion

It is clear that each of the proposed schemes can be divided into three distinct phases:preparing quantum channels, QT and the process of assisted quantum cloning. In our schemes, quantum channels prepared by entanglement compensation in AD noise are all PESs.In CQT, all participants, except for the state preparer, act as both transmitters and receivers of quantum information. During the cloning phase, however, these participants function solely as recipients of the replicated states. This structure ensures that the entire process follows a cyclical and synchronized pattern. Previous studies have found that: the fidelity and probability of quantum communication in a noisy environment are both less than 1 and decrease with the increase in noise. However, each of the protocols that we present in this paper demonstrates that the fidelity remains a constant 1, regardless of how the noise intensity of AD changes. Note that the success probabilities of the CQTs in the three-party schemes in Sects 3, 4 and 5 have the following relation:

$$\frac{8(1\;-\;\gamma {)}^{6}}{[1\;+\;(1\;-\;\gamma {)}^{2}{]}^{3}}>\frac{27(1\;-\;\gamma {)}^{6}}{[1\;+\;2(1\;-\;\gamma {)}^{2}{]}^{3}}>\frac{d^{3}(1\;-\;\gamma {)}^{6}}{[1\;+\;(d_{1})(1\;-\;\gamma {)}^{2}{]}^{3}}.$$ (96)

This means that the probability of success for the cyclic QT decreases as the dimensionality of the transmitted quantum states increases. Moreover, it can be known from the probability $8{\left(1\;-\;\gamma \right)}^{6}/[1\;+\;{\left(1\;-\;\gamma \right)}^{2}{]}^{3}$ of CQT in Sect 3.1 and the probability $2^{N}{\left(1\;-\;\gamma \right)}^{2N}/[1\;+\;{\left(1\;-\;\gamma \right)}^{2}{]}^{N}$ of CQT in Sect 3.2 that the more people involved in the communication, the smaller the probability of success of the CQT.

Introducing ancillary particles, implementing special unitary transformations, and performing single-particle *Z*-basis measurements in the CQTs of Sects 2, 4 and 5. A natural question is: Can positive-operator-valued measurement (POVM) be adopted? The answer is yes. To illustrate the problem we take the re-examination of Sect 3.1 (b3) as an example of applying the POVM method. For convenience of expression, let us define s⊕t=0. Then the state $[\alpha^{s⊕t⊕1}\beta^{s⊕t}|0\rangle \;+\;(1\;-\;\gamma )\alpha^{s⊕t}\beta^{s⊕t⊕1}|1\rangle {]}_{B}=[\alpha |0\rangle \;+\;(1\;-\;\gamma )\beta |1\rangle {]}_{B}$. After Bob introduces an auxiliary qubit *B*_2_ in initial state $|0\rangle_{B_{2}}$, he executes a CNOT gate

$$|G'''\rangle =N_{BB_{2}}([\alpha |0\rangle \;+\;(1\;-\;\gamma )\beta |1\rangle {]}_{B}|0\rangle_{B_{2}})/\sqrt{1\;+\;(1\;-\;\gamma {)}^{2}}\;=(\alpha |00\rangle \;+\;(1\;-\;\gamma )\beta |11\rangle {)}_{BB_{2}}/\sqrt{1\;+\;(1\;-\;\gamma {)}^{2}}\;=\frac{1}{2\sqrt{1\;+\;(1\;-\;\gamma {)}^{2}}}(|E\rangle_{B}⊗|F\rangle_{B_{2}}\;+\;|G\rangle_{B}⊗|H\rangle_{B_{4}}),$$ (97)

where $|E\rangle_{B}=(\alpha |0\rangle \;+\;\beta |1\rangle {)}_{B}$, $|F\rangle_{B_{2}}=[|0\rangle \;+\;(1\;-\;\gamma )|1\rangle {]}_{B_{2}}/\sqrt{1\;+\;{\left(1\;-\;\gamma \right)}^{2}}$, $|G\rangle_{B}=(\alpha |0\rangle \;-\;\beta |1\rangle {)}_{B}$ and $|H\rangle_{B_{2}}=[|0\rangle -(1\;-\;\gamma )|1\rangle {)}_{B_{2}}/\sqrt{1\;+\;{\left(1\;-\;\gamma \right)}^{2}}$.

In order to identify the states $|E\rangle_{B}$ and $|G\rangle_{B}$, Bob is required to perform a positive POVM on the auxiliary qubit *B*_2_. The corresponding POVM elements are constructed in the following form:

$$P_{1}=\frac{1}{\upsilon }|O_{1}\rangle \langle O_{1}|,\;P_{2}=\frac{1}{\upsilon }|O_{2}\rangle \langle O_{2}|,\;P_{3}=I-\frac{1}{\upsilon }\Sigma_{j=1}^{2}|O_{j}\rangle \langle O_{j}|,$$ (98)

here, the states are defined as $|O_{1}\rangle =\frac{1}{\sqrt{\varsigma }}\left[\sqrt{1\;+\;{\left(1\;-\;\gamma \right)}^{2}}|0\rangle \;+\;\frac{\sqrt{1\;+\;{\left(1\;-\;\gamma \right)}^{2}}}{1\;-\;\gamma }|1\rangle \right]B_{2}$ and $|O_{2}\rangle =\frac{1}{\sqrt{\varsigma }}\left[\sqrt{1\;+\;{\left(1\;-\;\gamma \right)}^{2}}|0\rangle -\frac{\sqrt{1\;+\;{\left(1\;-\;\gamma \right)}^{2}}}{1\;-\;\gamma }|1\rangle \right]B_{2}$, where the normalization factor is given by $\varsigma =\frac{[1\;+\;{\left(1\;-\;\gamma \right)}^{2}{]}^{2}}{{\left(1\;-\;\gamma \right)}^{2}}$. *I* denotes the identity operator. Additionally, the real parameter *υ*, which depends on *γ*, must be appropriately chosen to ensure that *P*_3_ is a positive operator.

In order to determine *υ*, we can rewrite $P_{1},P_{2}$ and *P*_3_ in the following matrix form

$$P_{1}=\frac{1}{\upsilon \varsigma }\left(\begin{array}{cc} 1\;+\;(1\;-\;\gamma {)}^{2} & \frac{1\;+\;(1\;-\;\gamma {)}^{2}}{1\;-\;\gamma }\\ \frac{1\;+\;(1\;-\;\gamma {)}^{2}}{1\;-\;\gamma } & \frac{1\;+\;(1\;-\;\gamma {)}^{2}}{(1\;-\;\gamma {)}^{2}} \end{array}\right),\;P_{2}=\frac{1}{\upsilon \varsigma }\left(\begin{array}{cc} 1\;+\;(1\;-\;\gamma {)}^{2} & -\frac{1\;+\;(1\;-\;\gamma {)}^{2}}{1\;-\;\gamma }\\ -\frac{1\;+\;(1\;-\;\gamma {)}^{2}}{1\;-\;\gamma } & \frac{1\;+\;(1\;-\;\gamma {)}^{2}}{(1\;-\;\gamma {)}^{2}} \end{array}\right),\;P_{3}=\left(\begin{array}{cc} 1-\frac{2(1\;-\;\gamma {)}^{2}}{\upsilon [1\;+\;(1\;-\;\gamma {)}^{2}]} & 0\\ 0 & 1-\frac{2}{\upsilon [1\;+\;(1\;-\;\gamma {)}^{2}]} \end{array}\right).$$ (99)

In order for *P*_3_ to be a positive operator, the parameter *υ* must satisfy the condition $\upsilon \geq \frac{2}{1\;+\;{\left(1-\gamma \right)}^{2}}$. Upon performing the POVM, Bob can obtain the measurement outcomes corresponding to *P*_1_ and *P*_2_ with the following probability:

$$p=\langle T_{2}^{\prime }|P_{1}|T_{2}^{\prime }\rangle =\langle T_{2}^{\prime }|P_{2}|T_{2}^{\prime }\rangle =\frac{1}{\upsilon \varsigma }=\frac{(1\;-\;\gamma {)}^{2}}{\upsilon [1\;+\;(1\;-\;\gamma {)}^{2}{]}^{2}},$$ (100)

and can infer the state of qubit *B*_2_ based on the POVM outcome.However, Bob is able to obtain the measurement outcome corresponding to the operator *P*_3_ with probability $1\;-\;\frac{2{\left(1\;-\;\gamma \right)}^{2}}{\upsilon [1\;+\;{\left(1\;-\;\gamma \right)}^{2}{]}^{2}}$, in which case he is unable to determine the specific state of the auxiliary particle *B*_2_. Conversely, if Bob successfully identifies that *B*_2_ is in the state $|F\rangle_{B_{2}}$ or $|H\rangle_{B_{2}}$, this implies that he knows the state of particle *B* is $|E\rangle_{B}$ or $|G\rangle_{B}$, respectively. Accordingly, he can apply the unitary operation $\sigma^{\left(0,0\right)}$ or $\sigma^{\left(1,1\right)}$ on qubit *B* to reconstruct the target state $|\xi \rangle_{A}$ on qubit *B* with a maximum success probability of ${\left(1\;-\;\gamma \right)}^{2}/2[1\;+\;{\left(1\;-\;\gamma \right)}^{2}]$. Obviously, both Charlie and Alice can reach the same conclusion.

A natural and critical question is whether similar probabilistic exact cloning protocols (with fidelity equal to 1) can be constructed under different noise environments, such as bit-flip or depolarizing noise. Here, we must emphasize that the protocol proposed in this paper is specifically designed for the AD noise model. The irreversible nature of AD noise, where the system spontaneously decays to its ground state, leads to directional evolution in quantum systems. This characteristic is crucial because the Kraus operator structure of AD noise enables selective measurement of the quantum system and post-projection onto the cloneable subspace. Based on this physical mechanism, our probabilistic post-selection cloning strategy is theorized, allowing us to construct a probabilistic cloning scheme with fidelity equal to 1. However, for most other types of noise models, such as bit-flip noise, the method proposed in this paper is not applicable, and the fidelity usually cannot reach 1. Similarly, in depolarizing noise environments, it is also difficult to achieve exact cloning. To determine whether our method is still feasible under a certain noise model, we provide a systematic verification method in the appendix. Specifically, the appendix presents the proof processes of two theorems, serving as the basis for judging different dimensional systems: Theorem 1 is designed for low-dimensional systems (such as single-qubit cases), and Theorem 2 extends to higher-dimensional systems (such as multi-level quantum systems). These theorems not only provide sufficient conditions for achieving perfect cloning but also offer a practical method to verify whether a specific noise model supports our protocol. Particularly, in the case of AD noise, both theorems are fully satisfied, thus proving the feasibility of our method in this environment. However, for most other models, these conditions are usually not met, rendering our method inapplicable. Future work could explore improved protocols for other noise channels.

Security is always a crucial consideration when choosing communication protocols. Below, we briefly discuss the security of the proposed scheme. In fact, the security of our scheme mainly depends on whether the quantum state, as a quantum communication channel, is securely shared by legitimate participants beforehand. In other words, the key is whether the entanglement is maintained securely during the distribution process. By employing existing, mature, and widely used entanglement detection methods [53,54] to preprocess the quantum entanglement distribution required in our scheme, we can effectively identify and defend against malicious attacks from external sources and fraudulent behaviors from internal sources. The specific detection process will not be elaborated on in this paper. This indicates that the safety of our scheme can be effectively guaranteed. Furthermore, the inclusion of a controller in the scheme enhances the overall security, ensuring that the proposed protocol is both secure and controllable.In conclusion, the proposed scheme is controllable and secure.

In summary, this paper explores the issue of conclusive cyclic assisted cloning of arbitrary unknown single-particle states within an AD channel, leveraging the technologies of QT and RSP. We focus on the scenario where the particles transmitted through the quantum channel experience the same independent AD strength, while the particles remaining locally are unaffected. The main contributions are as follows.

(i) The detailed process of sharing pure entangled single-qubit (single-qutrit) state among three communicators in AD channel is provided via CNOT gates (GCNOT gates). The method of sharing is easy generalize to the case of sharing an arbitrary *d*-dimensional pure single-qudit state.

(ii) Utilizing these shared pure single-particle states as the components of quantum channels, we present two three-party cyclic-assisted cloning protocols.Each protocol requires standard CQT and multi-output assisted cloning. In its CQT, the sender Alice teleports an arbitrary unknown single-qubit state (single-qutrit state) to Bob, Bob transmits an unknown single-qubit (single-qutrit) state to Charlie, and at the same time, Charlie also transmits an unknown single-qubit (single-qutrit) state to Alice. The probabilistic reconstruction of each of the three unknown quantum states is achieved by introducing an auxiliary qubit and performing suitable quantum operations. The success rate of this process depends solely on the smaller modulus of the coefficients that describe a particular shared PES. In the multi-output assisted cloning, after the special multi-particle measurement (unitary transformation and multi-particle equatorial state measurement) by the state preparer, the three original unknown are simultaneously cloned at three different locations with respective probabilities.

(iii) In the above two three-party cyclic-assisted cloning protocols, the analytical expressions of the operations of the participants we provided are as general as possible. This not only reveals the general rules more clearly and simplifies the cumbersome expressions, but also are easy to generalize to more general complex communication scenarios.

(iv) We have made general extensions of the above two three-party protocols from the two perspectives: the number of people participating in the circular communication and the dimension of the quantum system, which will be greatly beneficial to the future high-capacity quantum network communication.

(v) Although our scheme is based on the AD environment, its fidelities are all equal to 1, whereas almost all existing protocols cannot achieve such fidelity. Furthermore, a careful analysis of the scheme shows that in our protocols, complex multi-particle unitary matrices can be decomposed into simple 2×2 matrices, and that multi-particle measurements can be decomposed into single-particle projection measurements. Therefore, our protocol is closer to real life and has the potential to be experimentally realized as technology advances.

## Supporting information

S1 Fig Schematic diagram for three-party cyclic assisted cloning of single-qubit states in amplitude damping. (EPS)

S2 Fig Schematic diagram for multiparty cyclic assisted cloning of arbitrary single-qubit states in the amplitude damping channel. (EPS)

S1 Appendix Proof of Theorem 1. (PDF)

S2 Appendix Proof of Theorem 2. (PDF)

## References

[1] Bennett C, Brassard G, Crépeau C, Jozsa R, Peres A, Wootters W. Teleporting an unknown quantum state via dual classical and Einstein-Podolsky-Rosen channels. Phys Rev Lett. 1993;70(13):1895–9. doi: 10.1103/PhysRevLett.70.1895 10053414

[2] Liu B, Xia S, Xiao D, Huang W, Xu B, Li Y. Decoy-state method for quantum-key-distribution-based quantum private query. Sci China Phys Mech Astron. 2022;65(4):240312.doi: 10.1007/s11433-021-1843-7

[3] Gokul A, Natarajan H, Raghunathan V. Fiber-based higher dimensional quantum key distribution implementation using time-bin qudits. In: Quantum Computing, Communication, and Simulation IV. 2024. p. 112–9.

[4] Zhou L, Sheng Y-B. One-step device-independent quantum secure direct communication. Sci China Phys Mech Astron. 2022;65(5). doi: 10.1007/s11433-021-1863-9

[5] Li X-H. Quantum secure direct communication. Acta Phys Sin. 2015;64(16):160307. doi: 10.7498/aps.64.160307

[6] Shi G-F. Measurement-device-independent quantum dialogue. Chinese Phys B. 2021;30(10):100303. doi: 10.1088/1674-1056/ac140a

[7] Zhang C, Zhou L, Zhong W, Du M-M, Sheng Y-B. Measurement-device-independent quantum dialogue based on entanglement swapping and phase encoding. Quantum Inf Process. 2024;23(2):52.doi: 10.1007/s11128-024-04260-w

[8] Peng J, Maihemuti N, Aisan Y, Yang Z. Quantum teleportation of shared high-dimensional quantum secret. Phys Scr. 2024;99(8):085125. doi: 10.1088/1402-4896/ad623d

[9] Liu M, Maihemuti N, Peng J, Aisan Y, Tang J. Hierarchical quantum communication of a single-qubit state with multiple users. Laser Phys. 2025;35(4):045202. doi: 10.1088/1555-6611/adbad1

[10] Peng J, Liu M, Yang Z, Tang L, Tang J. Double-direction cyclic controlled quantum communication of single-particle states. Physica A: Statistical Mechanics and its Applications. 2023;632:129343. doi: 10.1016/j.physa.2023.129343

[11] Tang J, Maihemuti N, Peng J. Conclusive high-dimensional multiparty quantum state sharing in amplitude-damping channel. Int J Theor Phys. 2025;64(2):47.doi: 10.1007/s10773-025-05906-w

[12] Peng J-Y, Tang L, Yang Z. Deterministic hierarchical quantum operation sharing with five-qubit partially entangled states. Quantum Inf Process. 2023;22(7). doi: 10.1007/s11128-023-03963-w

[13] Sun S, Zhang H. Double-direction quantum cyclic controlled remote state preparation of two-qubit states. Quantum Inf Process. 2021;20(6). doi: 10.1007/s11128-021-03149-2

[14] Peng J-Y, Xiang Y. Multiparty-controlled remote state preparation of multiple qubit-string information. Int J Mod Phys B. 2023;38(14). doi: 10.1142/s0217979224501674

[15] Xin X, He S, Li C. Deterministic hierarchical joint remote state preparation via a non-maximally entangled state. Quantum Inf Process. 2024;23(4):121.doi: 10.1007/s11128-024-04329-6

[16] Maihemuti N, Chen Z, Peng J, Aisan Y, Tang J. Controlled double-direction cyclic quantum communication of arbitrary two-particle states. Entropy (Basel). 2025;27(3):292. doi: 10.3390/e27030292 40149216 PMC11941178

[17] Liu M, Maihemuti N, Peng J. Controlled bidirectional remote implementations of partially unknown quantum operations. Phys Scr. 2024;99(4):045109. doi: 10.1088/1402-4896/ad2d9d

[18] Zhang Z, Deng L, Zhang L, Zhuge B, Ye B. Efficient tripartite quantum operation sharing with five-qubit absolutely maximally entangled state. Int J Theor Phys. 2021;60(7):2583–91. doi: 10.1007/s10773-020-04684-x

[19] Wootters WK, Zurek WH. A single quantum cannot be cloned. Nature. 1982;299(5886):802–3. doi: 10.1038/299802a0

[20] Bužek V, Hillery M. Quantum copying: beyond the no-cloning theorem. Physical Review A. 1996;54(3):1844. 10.1103/physreva.54.1844 9913670

[21] Hillery M, Bužek V. Quantum copying: fundamental inequalities. Phys Rev A. 1997;56(2):1212–6. doi: 10.1103/physreva.56.1212

[22] Bužek V, Braunstein SL, Hillery M, Bruß D. Quantum copying: a network. Phys Rev A. 1997;56(5):3446–52. doi: 10.1103/physreva.56.3446

[23] Gisin N, Massar S. Optimal quantum cloning machines. Phys Rev Lett. 1997;79(11):2153–6. doi: 10.1103/physrevlett.79.2153

[24] Bruß D, DiVincenzo DP, Ekert A, Fuchs CA, Macchiavello C, Smolin JA. Optimal universal and state-dependent quantum cloning. Phys Rev A. 1998;57(4):2368–78. doi: 10.1103/physreva.57.2368

[25] Duan L-M, Guo G-C. A probabilistic cloning machine for replicating two non-orthogonal states. Physics Letters A. 1998;243(5–6):261–4. doi: 10.1016/s0375-9601(98)00287-4

[26] Duan L-M, Guo G-C. Probabilistic cloning and identification of linearly independent quantum states. Phys Rev Lett. 1998;80(22):4999–5002. doi: 10.1103/physrevlett.80.4999

[27] Murao M, Jonathan D, Plenio MB, Vedral V. Quantum telecloning and multiparticle entanglement. Phys Rev A. 1999;59(1):156–61. doi: 10.1103/physreva.59.156

[28] Bruß D, Cinchetti M, Mauro D, Ariano G. Phase covariant quantum cloning. Physical Review A. 2000;62(1):012302.

[29] Werner RF. Optimal cloning of pure states. Physical Review A. 1998;58(3):1827.

[30] Fan H. Quantum cloning of mixed states in symmetric subspace. Physical Review A. 2003;68(5):054301.

[31] Zou XB, Pahlke K, Mathis W. Scheme for the implementation of a universal quantum cloning machine via cavity-assisted atomic collisions in cavity QED. Physical Review A. 2003;67:024304.

[32] Pati AK. Assisted cloning and orthogonal complementing of an unknown state. Physical Review A. 2000;61(2):022308.

[33] Zhan Y-B. Assisted cloning of an unknown two-particle entangled state. Physics Letters A. 2005;336(4–5):317–23. doi: 10.1016/j.physleta.2004.12.073

[34] Han LF, Yuan H, Yang M, Cao ZL. Assisted cloning of an arbitrary unknown two-qubit state via a genuine four-qubit entangled state and positive operator-valued measure. Indian Journal of Pure & Applied Physics. 2014;52:563–70.

[35] Hou K, Shi SH. Scheme for cloning an unknown entangled state with assistance via non-maximally entangled cluster states. International Journal of Theoretical Physics. 2009, 48(1): 167–77.

[36] Briegel HJ, Raussendorf R. Persistent entanglement in arrays of interacting particles. Phys Rev Lett. 2001;86(5):910–3. doi: 10.1103/PhysRevLett.86.910 11177971

[37] Zhan Y. A scheme for assisted cloning of a two-particle entangled state via GHZ class states. Chin Opt Lett. 2005;3(101):S286–9.

[38] Ming F, Yi-Min L, Jun L, Shou-Hua S, Zhan-Jun Z. Assisted cloning and orthogonal complementing of an arbitrary unknown two-qubit entangled state. Commun Theor Phys. 2006;46(5):849–52. doi: 10.1088/0253-6102/46/5/016

[39] Peng-Cheng M, You-Bang Z. Scheme for implementing assisted cloning of an unknown d-dimension equatorial quantum state by remote state preparation. Commun Theor Phys. 2009;51(1):57–9. doi: 10.1088/0253-6102/51/1/11

[40] Chen XB, Ma SY, Pang B, Yang YX, Wen QY. An economical scheme for cloning an unknown M-qudit equatorial-like state with assistance. Communications in Theoretical Physics. 2010;53(6):1067–71.

[41] Lipeng Xue, Min Jiang. Assisted cloning of arbitrary unknown multi-qudit state. In: 2015 10th Asian Control Conference (ASCC). 2015. p. 1–5. 10.1109/ascc.2015.7244542

[42] Maihemuti N, Peng J, Yang Z, Zhai D, Tang J. Controlled and assisted cloning of an arbitrary unknown states via maximal slice states. Int J Theor Phys. 2024;63(1). doi: 10.1007/s10773-023-05536-0

[43] Zhai D, Peng J, Maihemuti N, Tang J. Assisted cloning of an arbitrary unknown d-dimension state. Int J Theor Phys. 2024;63(6). doi: 10.1007/s10773-024-05694-9

[44] Yang G, Lian B-W, Nie M, Jin J. Bidirectional multi-qubit quantum teleportation in noisy channel aided with weak measurement. Chinese Phys B. 2017;26(4):040305. doi: 10.1088/1674-1056/26/4/040305

[45] Peng J-Y, Xiang Y. Bidirectional remote state preparation in noisy environment assisted by weak measurement. Optics Communications. 2021;499:127285. doi: 10.1016/j.optcom.2021.127285

[46] Peng J-Y, Tang L, Yang Z, Lei H, Bai M-Q. Many-party controlled remote implementations of multiple partially unknown quantum operations. Quantum Inf Process. 2022;22(1). doi: 10.1007/s11128-022-03750-z

[47] Sun S, Zhang H. Deterministic quantum cyclic controlled teleportation of arbitrary multi-qubit states using multi-qubit partially entangled channel. Mod Phys Lett A. 2020;35(25):2050204. doi: 10.1142/s0217732320502041

[48] Jia-yin P, Hong-xuan L. Cyclic remote state preparation. Int J Theor Phys. 2021;60(4):1593–602. doi: 10.1007/s10773-021-04782-4

[49] Sun S, Zhang H. Quantum double-direction cyclic controlled communication via a thirteen-qubit entangled state. Quantum Inf Process. 2020;19(4):1–18.doi: 10.1007/s11128-020-2619-5

[50] Peng J, Yang Z, Tang L, Peng J. Double-direction cyclic controlled remote implementation of partially known quantum operations. Int J Theor Phys. 2022;61(10). doi: 10.1007/s10773-022-05213-8

[51] Hu WW, Zhou R-G, Luo GF. Conclusive multiparty quantum state sharing in amplitude-damping channel. Quantum Inf Process. 2021;21(1). doi: 10.1007/s11128-021-03333-4

[52] Fonseca A. High-dimensional quantum teleportation under noisy environments. Physical Review A. 2019;100(6):062311.

[53] Deng FG, Li XH, Zhou HY, et al. Improving the security of multiparty quantum secret sharing against Trojan horse attack. Physical Review A 2005;72:044302.

[54] Zhang ZJ, Yang J, Man ZX, Li Y. Multiparty secret sharing of quantum information using and identifying Bell states. Eur Phys J D. 2005;33(1):133–6. doi: 10.1140/epjd/e2005-00029-5

