# Supplementary material for: Cyclic assisted cloning of arbitrary unknown single-particle states in amplitude damping channel
Source: PLoS One. 2025 Sep 2;20(9):e0329370. doi: 10.1371/journal.pone.0329370 (PMC12404563; doi:10.1371/journal.pone.0329370)
Supplement: S1 Appendix — (PDF) [file pone.0329370.s003.pdf]

S1 Appendix Proof of Theorem 1

Theorem 1 On the condition that the Bell state $|\mathcal{B}_{00}\rangle = (|00\rangle + |11\rangle)_{AA_1}/\sqrt{2}$ of two qubits A and A_1 is prepared by Alice, when she measures qubit A_1 in the computational basis $\{|0\rangle, |1\rangle\}$ and obtains the result $|0\rangle_{A_1}$ as described in (a3) of Section 3.1, Alice will then share the following PES

$$|\mathcal{H}\rangle_{AB} = \frac{1}{\sqrt{1+(1-\gamma)^2}}[|00\rangle + (1-\gamma)|11\rangle]_{AB}$$

with Bob.

Proof 1 After Alice conveys the qubit A_1 to Bob through the AD channel, the density matrix of the entangled state composed (DMESC) of qubit pair (A, A_1) can be expressed as:

$$\begin{aligned}\rho_b = \varepsilon(\rho) &= \sum_{j=0}^{1}(I \otimes K_j) * \rho * (I \otimes K_j)^\dagger \\ &= \frac{1}{2}[|00\rangle\langle 00| + \sqrt{1-\gamma}|00\rangle\langle 11| + \sqrt{\gamma}|00\rangle\langle 10| \\ &+ \sqrt{1-\gamma}|11\rangle\langle 00| + \sqrt{\gamma}|10\rangle\langle 00| + (1-\gamma)|11\rangle\langle 11| \\ &+ \sqrt{(1-\gamma)\gamma}|11\rangle\langle 10| + \sqrt{\gamma(1-\gamma)}|10\rangle\langle 11| + \gamma|10\rangle\langle 10|],\end{aligned} \tag{1}$$

where $\rho = |\mathcal{B}_{00}\rangle_{AA_1}\langle\mathcal{B}_{00}|$, and ρ_b represents DMESC composed of qubit pair (A, A_1) when Bob receives the qubit A_1.

When Bob applies a CNOT gate on qubit A_1 and auxiliary qubit B with the initial state $|0\rangle_B$, where qubits A_1 and B act as the control and target qubits, respectively, the density matrix of the state composed of qubit group (A, A_1, B) is written as:

$$\begin{aligned}\rho_{b^*} &= \frac{1}{2}[|000\rangle\langle 000| + \sqrt{1-\gamma}|000\rangle\langle 111| + \sqrt{\gamma}|000\rangle\langle 100| \\ &+ \sqrt{1-\gamma}|111\rangle\langle 000| + \sqrt{\gamma}|100\rangle\langle 000| + (1-\gamma)|111\rangle\langle 111| \\ &+ \sqrt{(1-\gamma)\gamma}|111\rangle\langle 100| + \sqrt{\gamma(1-\gamma)}|100\rangle\langle 111| + \gamma|100\rangle\langle 100|].\end{aligned} \tag{2}$$

Subsequently, Bob transmits qubit A_1 to Alice via AD channel. After Alice gets the qubit A_1, the density matrix of the entangled state consisting of qubit group (A, A_1, B) evolves into:

$$\begin{aligned}\rho_a = \varepsilon(\rho_{b^*}) &= \sum_{j=0}^{1}(I \otimes K_j \otimes I) * \rho_{b^*} * (I \otimes K_j \otimes I)^\dagger \\ &= \frac{1}{2}[|000\rangle\langle 000| + (1-\gamma)|000\rangle\langle 111| + \sqrt{(1-\gamma)\gamma}|000\rangle\langle 101| \\ &+ \sqrt{\gamma}|000\rangle\langle 100| + (1-\gamma)|111\rangle\langle 000| + \sqrt{(1-\gamma)\gamma}|101\rangle\langle 000| \\ &+ \sqrt{\gamma}|100\rangle\langle 000| + (1-\gamma)^2|111\rangle\langle 111| + \sqrt{(1-\gamma)^3\gamma}|111\rangle\langle 101| \\ &+ \sqrt{(1-\gamma)^3\gamma}|101\rangle\langle 111| + (1-\gamma)\gamma|101\rangle\langle 101| + (1-\gamma)\sqrt{\gamma}|111\rangle\langle 100| \\ &+ \sqrt{(1-\gamma)}\gamma|101\rangle\langle 100| + (1-\gamma)\sqrt{\gamma}|100\rangle\langle 111| \\ &+ \gamma\sqrt{(1-\gamma)}|100\rangle\langle 101| + \gamma|100\rangle\langle 100|].\end{aligned} \tag{3}$$

Final, Alice first executes the CNOT operation on qubits A and A_1, where qubit A works as the control qubit

and A_1 as the target qubit, DMESC of qubit group (A, A_1, B) is written as:

$$\begin{aligned}
\rho_{a^*} = & \frac{1}{2}[|000\rangle\langle 000| + (1-\gamma)|000\rangle\langle 101| + \sqrt{(1-\gamma)\gamma}|000\rangle\langle 111| \\
& + \sqrt{\gamma}|000\rangle\langle 110| + (1-\gamma)|101\rangle\langle 000| + \sqrt{(1-\gamma)\gamma}|111\rangle\langle 000| \\
& + \sqrt{\gamma}|110\rangle\langle 000| + (1-\gamma)^2|101\rangle\langle 101| + \sqrt{(1-\gamma)^3\gamma}|101\rangle\langle 111| \\
& + \sqrt{(1-\gamma)^3\gamma}|111\rangle\langle 101| + (1-\gamma)\gamma|111\rangle\langle 111| + (1-\gamma)\sqrt{\gamma}|101\rangle\langle 110| \\
& + \sqrt{(1-\gamma)}\gamma|111\rangle\langle 110| + (1-\gamma)\sqrt{\gamma}|110\rangle\langle 101| \\
& + \gamma\sqrt{(1-\gamma)}|110\rangle\langle 111| + \gamma|110\rangle\langle 110|].
\end{aligned} \tag{4}$$

Therefore, when Alice makes a single-qubit projective measurement on qubit A_1 in the computational basis $\{|0\rangle, |1\rangle\}$, qubit A_1 is released from entanglement with the other three qubits (A, B, C). If the measurement outcome is $|0\rangle_{A_1}$,the DMESC of qubit group (A, B, C) collapses into:

$$\begin{aligned}
\rho_{AB} &= \frac{1}{2}[|00\rangle\langle 00| + (1-\gamma)|00\rangle\langle 11| + (1-\gamma)|11\rangle\langle 00| + (1-\gamma)^2|11\rangle\langle 11|] \\
&= \frac{1}{\sqrt{2}}[|00\rangle + (1-\gamma)|11\rangle]\frac{1}{\sqrt{2}}[\langle 00| + (1-\gamma)\langle 11|].
\end{aligned} \tag{5}$$

After renormalization of ρ_{AB}, Alice shares the following PES

$$|\mathcal{H}\rangle_{AB} = \frac{1}{\sqrt{1+(1-\gamma)^2}}[|00\rangle + (1-\gamma)|11\rangle]_{AB} \tag{6}$$

with Bob.
