# Supplementary material for: Cyclic assisted cloning of arbitrary unknown single-particle states in amplitude damping channel
Source: PLoS One. 2025 Sep 2;20(9):e0329370. doi: 10.1371/journal.pone.0329370 (PMC12404563; doi:10.1371/journal.pone.0329370)
Supplement: S2 Appendix — (PDF) [file pone.0329370.s004.pdf]

S2 Appendix Proof of Theorem 2

Theorem 2 Given that Alice prepares the generalized Bell state $|\mathcal{GB}00\rangle = (|00\rangle+|11\rangle+|22\rangle)AA_1/\sqrt{3}$ involving two qutrits A and A_1, a projective measurement is performed on qutrit A_1 in the computational basis $|0\rangle, |1\rangle, |2\rangle$.Alice shares the following PES:

$$|\mathcal{H}'\rangle_{AB} = \frac{1}{\sqrt{1+2(1-\gamma)^2}}[|00\rangle + (1-\gamma)|11\rangle + (1-\gamma)|22\rangle]_{AB}$$

with Bob.

Proof 2 After Alice conveys the qutrit A_1 to Bob via AD channel, DMESC of qutrit pair (A, A_1) is given by:

$$\begin{aligned}
\rho_{b'} &= \varepsilon(\rho') = \sum_{j=0}^{2}(I \otimes F_j) * \rho' * (I \otimes F_j)^\dagger \\
&= \frac{1}{3}[|00\rangle\langle 00| + \sqrt{1-\gamma}|00\rangle\langle 11| + \sqrt{\gamma}|00\rangle\langle 10| \\
&+ \sqrt{1-\gamma}|00\rangle\langle 22| + \sqrt{\gamma}|00\rangle\langle 20| + \sqrt{1-\gamma}|11\rangle\langle 00| \\
&+ \sqrt{\gamma}|10\rangle\langle 00| + (1-\gamma)|11\rangle\langle 11| + \sqrt{(1-\gamma)\gamma}|11\rangle\langle 10| \\
&+ \sqrt{(1-\gamma)\gamma}|10\rangle\langle 11| + \gamma|10\rangle\langle 10| + (1-\gamma)|11\rangle\langle 22| \\
&+ \sqrt{(1-\gamma)\gamma}|11\rangle\langle 20| + \sqrt{(1-\gamma)\gamma}|10\rangle\langle 22| + \gamma|10\rangle\langle 20| \\
&+ \sqrt{1-\gamma}|22\rangle\langle 00| + \sqrt{\gamma}|20\rangle\langle 00| + (1-\gamma)|22\rangle\langle 11| \\
&+ \sqrt{(1-\gamma)\gamma}|22\rangle\langle 10| + \sqrt{(1-\gamma)\gamma}|20\rangle\langle 11| + \gamma|20\rangle\langle 10| \\
&+ (1-\gamma)|22\rangle\langle 22| + \sqrt{(1-\gamma)\gamma}|22\rangle\langle 20| + \sqrt{(1-\gamma)\gamma}|20\rangle\langle 22| \\
&+ \gamma|20\rangle\langle 20|],
\end{aligned} \tag{1}$$

where $\rho' = |\mathcal{GB}_{00}\rangle_{AA_1}\langle\mathcal{GB}_{00}|$, and $\rho_{b'}$ denotes DMESC of qutrit pair (A, A_1) when Bob gets the qutrit A_1.

When Bob executes a generalized CNOT gate on qutrit A_1 and auxiliary qutrit B with initial state $|0\rangle_B$, where qutrits A_1 and B works as the control and garget qutrits respectively, the density matrix of the state composed of qutrit group (A, A_1, B) is written as:

$$\begin{aligned}
\rho^*_{b'} &= \frac{1}{3}[|000\rangle\langle 000| + \sqrt{1-\gamma}|000\rangle\langle 111| + \sqrt{\gamma}|000\rangle\langle 100| \\
&+ \sqrt{1-\gamma}|000\rangle\langle 222| + \sqrt{\gamma}|000\rangle\langle 200| + \sqrt{1-\gamma}|111\rangle\langle 000| \\
&+ \sqrt{\gamma}|100\rangle\langle 000| + (1-\gamma)|111\rangle\langle 111| + \sqrt{(1-\gamma)\gamma}|111\rangle\langle 100| \\
&+ \sqrt{(1-\gamma)\gamma}|100\rangle\langle 111| + \gamma|100\rangle\langle 100| + (1-\gamma)|111\rangle\langle 222| \\
&+ \sqrt{(1-\gamma)\gamma}|111\rangle\langle 200| + \sqrt{(1-\gamma)\gamma}|100\rangle\langle 222| + \gamma|100\rangle\langle 200| \\
&+ \sqrt{1-\gamma}|222\rangle\langle 000| + \sqrt{\gamma}|200\rangle\langle 000| + (1-\gamma)|222\rangle\langle 111| \\
&+ \sqrt{(1-\gamma)\gamma}|222\rangle\langle 100| + \sqrt{(1-\gamma)\gamma}|200\rangle\langle 111| + \gamma|200\rangle\langle 100| \\
&+ (1-\gamma)|222\rangle\langle 222| + \sqrt{(1-\gamma)\gamma}|222\rangle\langle 200| + \sqrt{(1-\gamma)\gamma}|200\rangle\langle 222| \\
&+ \gamma|200\rangle\langle 200|].
\end{aligned} \tag{2}$$

Subsequently, Bob transmits qutrit A_1 to Alice via AD channel. After Alice gets the qutrit A_1, DMESC of qutrit

group (A, A_1, B) evolves into:

$$\begin{aligned}
\rho_{a'} &= \varepsilon(\rho^*_{b'}) = \sum_{j=0}^{2}(I \otimes F_j \otimes I) * \rho^*_{b'} * (I \otimes F_j \otimes I)^\dagger \\
&= \frac{1}{3}[|000\rangle\langle 000| + (1-\gamma)|000\rangle\langle 111| + \sqrt{(1-\gamma)\gamma}|000\rangle\langle 101| \\
&+ \sqrt{\gamma}|000\rangle\langle 100| + (1-\gamma)|000\rangle\langle 222| + \sqrt{(1-\gamma)\gamma}|000\rangle\langle 202| \\
&+ \sqrt{\gamma}|000\rangle\langle 200| + (1-\gamma)|111\rangle\langle 000| + \sqrt{(1-\gamma)\gamma}|101\rangle\langle 000| \\
&+ \sqrt{\gamma}|100\rangle\langle 000| + (1-\gamma)^2|111\rangle\langle 111| + \sqrt{(1-\gamma)^3\gamma}|111\rangle\langle 101| \\
&+ \sqrt{(1-\gamma)^3\gamma}|101\rangle\langle 111| + (1-\gamma)\gamma|101\rangle\langle 101| + (1-\gamma)\sqrt{\gamma}|111\rangle\langle 100| \\
&+ \sqrt{(1-\gamma)\gamma}|101\rangle\langle 100| + (1-\gamma)\sqrt{\gamma}|100\rangle\langle 111| + \sqrt{(1-\gamma)\gamma}|100\rangle\langle 101| \\
&+ \gamma|100\rangle\langle 100| + (1-\gamma)^2|111\rangle\langle 222| + \sqrt{(1-\gamma)^3\gamma}|111\rangle\langle 202| \\
&+ \sqrt{(1-\gamma)^3\gamma}|101\rangle\langle 222| + (1-\gamma)\gamma|101\rangle\langle 202| + (1-\gamma)\sqrt{\gamma}|111\rangle\langle 200| \\
&+ \sqrt{(1-\gamma)\gamma}|101\rangle\langle 200| + (1-\gamma)\sqrt{\gamma}|100\rangle\langle 222| + \sqrt{(1-\gamma)\gamma}|100\rangle\langle 202| \\
&+ \gamma|100\rangle\langle 200| + (1-\gamma)|222\rangle\langle 000| + \sqrt{(1-\gamma)\gamma}|202\rangle\langle 000| \\
&+ \sqrt{\gamma}|200\rangle\langle 000| + (1-\gamma)^2|222\rangle\langle 111| + \sqrt{(1-\gamma)^3\gamma}|222\rangle\langle 101| \\
&+ \sqrt{(1-\gamma)^3\gamma}|202\rangle\langle 111| + (1-\gamma)\gamma|202\rangle\langle 101| + (1-\gamma)\sqrt{\gamma}|222\rangle\langle 100| \\
&+ \sqrt{(1-\gamma)\gamma}|202\rangle\langle 100| + (1-\gamma)\sqrt{\gamma}|200\rangle\langle 111| + \sqrt{(1-\gamma)\gamma}|200\rangle\langle 101| \\
&+ \gamma|200\rangle\langle 100| + (1-\gamma)^2|222\rangle\langle 222| + \sqrt{(1-\gamma)^3\gamma}|222\rangle\langle 202| \\
&+ \sqrt{(1-\gamma)^3\gamma}|202\rangle\langle 222| + (1-\gamma)\gamma|202\rangle\langle 202| + (1-\gamma)\sqrt{\gamma}|222\rangle\langle 200| \\
&+ \sqrt{(1-\gamma)\gamma}|202\rangle\langle 200| + (1-\gamma)\sqrt{\gamma}|200\rangle\langle 222| \\
&+ \sqrt{(1-\gamma)\gamma}|200\rangle\langle 202| + \gamma|200\rangle\langle 200|].
\end{aligned} \tag{3}$$

Finally, Alice first performs the IGCNOT operation on the qutrit pair (A, A_1), where qutrit A acts as the control qutrit and A_1 as the target qutrit. Then, the DMESC of the qutrit group (A, A_1, B) is written as:

$$\begin{aligned}
\rho^*_{a'} &= \frac{1}{3}[|000\rangle\langle 000| + (1-\gamma)|000\rangle\langle 101| + \sqrt{(1-\gamma)\gamma}|000\rangle\langle 111| \\
&+ \sqrt{\gamma}|000\rangle\langle 110| + (1-\gamma)|000\rangle\langle 202| + \sqrt{(1-\gamma)\gamma}|000\rangle\langle 222| \\
&+ \sqrt{\gamma}|000\rangle\langle 220| + (1-\gamma)|101\rangle\langle 000| + \sqrt{(1-\gamma)\gamma}|111\rangle\langle 000| \\
&+ \sqrt{\gamma}|110\rangle\langle 000| + (1-\gamma)^2|101\rangle\langle 101| + \sqrt{(1-\gamma)^3\gamma}|101\rangle\langle 111| \\
&+ \sqrt{(1-\gamma)^3\gamma}|111\rangle\langle 101| + (1-\gamma)\gamma|111\rangle\langle 111| + (1-\gamma)\sqrt{\gamma}|101\rangle\langle 110|
\end{aligned}$$

$$
\begin{aligned}
&+\sqrt{(1-\gamma)\gamma}|111\rangle\langle 110| + (1-\gamma)\sqrt{\gamma}|110\rangle\langle 101| + \sqrt{(1-\gamma)\gamma}|110\rangle\langle 111| \\
&+\gamma|110\rangle\langle 110| + (1-\gamma)^2|101\rangle\langle 202| + \sqrt{(1-\gamma)^3\gamma}|101\rangle\langle 222| \\
&+\sqrt{(1-\gamma)^3\gamma}|111\rangle\langle 202| + (1-\gamma)\gamma|111\rangle\langle 222| + (1-\gamma)\sqrt{\gamma}|101\rangle\langle 220| \\
&+\sqrt{(1-\gamma)\gamma}|111\rangle\langle 220| + (1-\gamma)\sqrt{\gamma}|110\rangle\langle 202| + \sqrt{(1-\gamma)\gamma}|110\rangle\langle 222| \\
&+\gamma|110\rangle\langle 220| + (1-\gamma)|202\rangle\langle 000| + \sqrt{(1-\gamma)\gamma}|222\rangle\langle 000| \\
&+\sqrt{\gamma}|220\rangle\langle 000| + (1-\gamma)^2|202\rangle\langle 101| + \sqrt{(1-\gamma)^3\gamma}|202\rangle\langle 111| \\
&+\sqrt{(1-\gamma)^3\gamma}|222\rangle\langle 101| + (1-\gamma)\gamma|222\rangle\langle 111| + (1-\gamma)\sqrt{\gamma}|202\rangle\langle 110| \\
&+\sqrt{(1-\gamma)\gamma}|222\rangle\langle 110| + (1-\gamma)\sqrt{\gamma}|220\rangle\langle 101| + \sqrt{(1-\gamma)\gamma}|220\rangle\langle 111| \\
&+\gamma|220\rangle\langle 110| + (1-\gamma)^2|202\rangle\langle 202| + \sqrt{(1-\gamma)^3\gamma}|202\rangle\langle 222| \\
&+\sqrt{(1-\gamma)^3\gamma}|222\rangle\langle 202| + (1-\gamma)\gamma|222\rangle\langle 222| + (1-\gamma)\sqrt{\gamma}|202\rangle\langle 220| \\
&+\sqrt{(1-\gamma)\gamma}|222\rangle\langle 220| + (1-\gamma)\sqrt{\gamma}|220\rangle\langle 202| \\
&+\sqrt{(1-\gamma)\gamma}|220\rangle\langle 222| + \gamma|220\rangle\langle 220|].
\end{aligned} \tag{4}
$$

Therefore, when Alice performs a single-qutrit projective measurement on qutrit A_1 in the computational basis $\{|0\rangle, |1\rangle, |2\rangle\}$, qutrit A_1 is disentangled from the remaining three qutrits (A, A_1, B).If the result of the measurement is $|0\rangle_{A_1}$, the DMESC of qutrit pair (A, B) collapses into

$$
\begin{aligned}
\rho_{AB} = &\frac{1}{3}[|00\rangle\langle 00| + (1-\gamma)|00\rangle\langle 11| + (1-\gamma)|00\rangle\langle 22| \\
&+ (1-\gamma)|11\rangle\langle 00| + (1-\gamma)^2|11\rangle\langle 11| + (1-\gamma)^2|11\rangle\langle 22| \\
&+ (1-\gamma)|22\rangle\langle 00| + (1-\gamma)^2|22\rangle\langle 11| + (1-\gamma)^2|22\rangle\langle 22|] \\
= &\frac{1}{\sqrt{3}}[|00\rangle + (1-\gamma|11\rangle + (1-\gamma)|22\rangle] \\
&\otimes \frac{1}{\sqrt{3}}[\langle 00| + (1-\gamma)\langle 11| + (1-\gamma)\langle 22|].
\end{aligned} \tag{5}
$$

After renormalization of ρ_{AB}, Alice shares the following PES

$$
|\mathcal{H}'\rangle_{AB} = \frac{1}{\sqrt{1+2(1-\gamma)^2}}[|00\rangle + (1-\gamma)|11\rangle + (1-\gamma)|22\rangle]_{AB} \tag{6}
$$

with Bob.
